# P1 Ref Endonuclease: A Molecular Mechanism for Phage-Enhanced Antibiotic Lethality

**DOI:** 10.1371/journal.pgen.1005797

**Published:** 2016-01-14

**Authors:** Erin A. Ronayne, Y. C. Serena Wan, Beth A. Boudreau, Robert Landick, Michael M. Cox

**Affiliations:** 1 Department of Biochemistry, University of Wisconsin-Madison, Madison, Wisconsin, United States of America; 2 Department of Bacteriology, University of Wisconsin-Madison, Madison, Wisconsin, United States of America; Université Paris Descartes, INSERM U1001, FRANCE

## Abstract

Ref is an HNH superfamily endonuclease that only cleaves DNA to which RecA protein is bound. The enigmatic physiological function of this unusual enzyme is defined here. Lysogenization by bacteriophage P1 renders *E*. *coli* more sensitive to the DNA-damaging antibiotic ciprofloxacin, an example of a phenomenon termed phage-antibiotic synergy (PAS). The complementary effect of phage P1 is uniquely traced to the P1-encoded gene *ref*. Ref is a P1 function that amplifies the lytic cycle under conditions when the bacterial SOS response is induced due to DNA damage. The effect of Ref is multifaceted. DNA binding by Ref interferes with normal DNA metabolism, and the nuclease activity of Ref enhances genome degradation. Ref also inhibits cell division independently of the SOS response. Ref gene expression is toxic to *E*. *coli* in the absence of other P1 functions, both alone and in combination with antibiotics. The RecA proteins of human pathogens *Neisseria gonorrhoeae* and *Staphylococcus aureus* serve as cofactors for Ref-mediated DNA cleavage. Ref is especially toxic during the bacterial SOS response and the limited growth of stationary phase cultures, targeting aspects of bacterial physiology that are closely associated with the development of bacterial pathogen persistence.

## Author Summary

The bacteriophage P1-encoded Ref protein is a novel class of endonuclease, cleaving only DNA to which the bacterial RecA protein is bound. The physiological role of such an enzyme has been entirely enigmatic. In this article, we define Ref as a P1 function that amplifies the lytic cycle under conditions when the bacterial SOS response is induced due to DNA damage. DNA-damaging antibiotics such as ciprofloxacin are much more lethal in bacteriophage P1 lysogens, and Ref is uniquely responsible for the complementation. In a phenomenon related to reported examples of phage-antibiotic synergy, Ref is highly toxic when expressed in bacteria in the absence of other P1 functions. Ref cleaves DNA in partnership with the RecA proteins from at least two prominent pathogens. The unstructured N-terminus of Ref also binds to DNA and interferes with normal DNA metabolism. When combined with antibiotics such as ciprofloxacin, Ref targets the bacterial functions most closely associated with bacterial persistence.

## Introduction

As multidrug resistance in important bacterial pathogens becomes an ever more serious health crisis [1], a need for new therapeutic strategies is evident [2–5]. An older approach, abandoned in the west in the 1940s but generating renewed interest, is phage therapy, as recently reviewed [6]. Phage therapy is slowly advancing as an approach to improve food safety, enhance water quality, provide an alternative to antibiotics in food-producing animals, facilitate environmental biocontrol of multidrug resistant pathogens on surfaces in hospitals, and treat wounds in humans [7]. Currently, phage therapy is approved in the United States only for control of pathogens in food sources and plants [8, 9].

Phage therapy may prove useful, not just as an alternative to antibiotics, but also as a complement. Phage-antibiotic synergy or PAS—has been noted in multiple reports involving a variety of bacterial species [10–15]. Although the degrees of complementarity vary, the phenomenon does not typically reflect synergy in the genetic sense; i.e., where the effects of the phage and antibiotic are reliably greater than additive. The established definition of PAS is a phenomenon whereby sub-lethal concentrations of certain antibiotics can substantially stimulate the host bacteria’s production of virulent phage [15]. The overall result is a complementation of antibiotic action by phage in reducing bacterial survival.

Whereas PAS, or in general a combination of antibiotic and phage therapy, represents a promising alternative therapeutic avenue for multidrug resistant pathogen infections, progress is constrained in part by a lack of understanding of the molecular mechanism(s) underlying the phage contribution. In this report, we explore the molecular basis of PAS using a classical phage system, the bacteriophage P1 of *Escherichia coli*. In this case, we show that the origin of an observed PAS phenomenon can be traced to the activity of a single P1-encoded gene, called *ref* for *r*ecombination *e*nhancement *f*unction [16–18].

First described in 1951 [19], bacteriophage P1 remains a mainstay of bacterial molecular biology. Unlike many other temperate phage, the P1 genome (93.6 kbp; ~117 genes) is not integrated into the host chromosome, but instead is maintained as a low copy number, autonomous plasmid [20]. As a lysogen, the C1 repressor prevents expression of P1 lytic genes [21]. When the host cell is placed under stress, the phage P1 lytic cycle is induced [22–24]. As defined in the current study, part of that cycle involves the P1 RecA-dependent nuclease Ref.

The Ref protein is an HNH-superfamily endonuclease (named for Histidine-Asparagine-Histidine, the three catalytic residues), with the novel property that it will only cleave DNA to which bacterial RecA protein is bound [25]. The Ref protein has a globular C-terminal domain that includes the nuclease active site [25]. The unstructured and highly charged N-terminal domain (76 amino acid residues) is required for single-strand and double-strand DNA binding [26]. Ref cleaves single-stranded DNA bound by RecA. It will also cleave both strands of the target duplex in DNA displacement loops (D-loops) that are created by RecA [25–27]. Both forms of RecA-bound DNA are found in bacterial cells, but become particularly numerous when the cell’s DNA is damaged. The nuclease active site resides in the C-terminal domain and consists of three histidine residues that coordinate a Zn^2+^ ion [25]. Changing one histidine (His 153) to alanine eliminates almost all of the nuclease activity [25, 27]. Based on HNH nuclease precedent [28], we have recently constructed a mutant converting two Zn^2+^ coordination residues (His 153 and His 134) to alanine to eliminate Zn^2+^ binding and completely inactivate nuclease activity.

In spite of the recent advances in understanding Ref biochemistry *in vitro*, the *in vivo* role of the Ref endonuclease has not been addressed. Expression of Ref in *E*. *coli* enhances some classes of recombination events in a RecA- and RecBCD-dependent manner [16, 17, 29]. Deletion of the *ref* gene in bacteriophage P1 had no apparent effect on lysogenic or lytic cycles in early reports [17, 18]. The RecA-dependent endonuclease function of Ref was unexpected [25], and the activity remains both unprecedented and perplexing. The current study began as an attempt to understand why an enzyme like Ref would evolve and be maintained by a bacteriophage.

In addition to its Ref-related activity, the bacterial RecA recombinase plays multiple roles in the maintenance of bacterial genome stability [30–35]. RecA forms nucleoprotein filaments on single-stranded DNA, and functions directly in all recombination processes via its DNA pairing and strand exchange activities [36–41]. RecA also plays a pivotal role in induction of the SOS response, by cleaving the bacterial LexA repressor in response to DNA damage. This results in the expression of a large number of genes that are directly or indirectly involved in DNA repair [42–44]. An example of an indirect effect on DNA repair can be seen in the SOS-inducible gene *sulA*, the product of which interacts with FtsZ to prevent formation of the Z-ring and delay cell division until the genome is faithfully repaired [45, 46]. Individual cells in a bacterial population that induce the SOS response or are otherwise in a slow growth state sometimes survive antibiotic treatment [47]. The SOS response has thus drawn attention as a potential therapeutic target in addressing multidrug resistance and its genesis [48–54].

There are no bacterial proteins more central to SOS induction than the RecA protein. This in turn provides a rationale for the evolution of a bacteriophage function that interacts with RecA. To enhance its lytic cycle under conditions of cellular stress, the bacteriophage P1 targets the SOS response with Ref: binding DNA, cleaving genomic DNA to which the RecA protein is bound, and causing cell filamentation. As we demonstrate here, these activities substantially enhance the lethality of antimicrobial agents that damage DNA and induce SOS. Ref is most toxic to bacteria in the precise situations where antibiotics are least toxic: during SOS and stationary phase; making it a potential candidate for combination phage/antibiotic therapeutic regimens.

## Results

There were two goals to this study. We wished to determine the physiological function of the P1-encoded Ref endonuclease, and also wished to explore the effects of this enzyme when expressed in *E*. *coli* in the absence of other phage factors. We first explore an example of enhancement of antibiotic activity by bacteriophage P1. We then define the function of the *ref* gene in the P1 life cycle, and finally demonstrate that the *ref* gene is both required and sufficient to account for the phage-mediated enhancement of antibiotic lethality in the case of bacteriophage P1. Physiological effects of both the nuclease catalytic domain of Ref and the unstructured N-terminal DNA binding domain of Ref are defined.

### In *Escherichia coli*, bacteriophage P1 lysogens enhance the toxicity of ciprofloxacin

**Fig 1:**
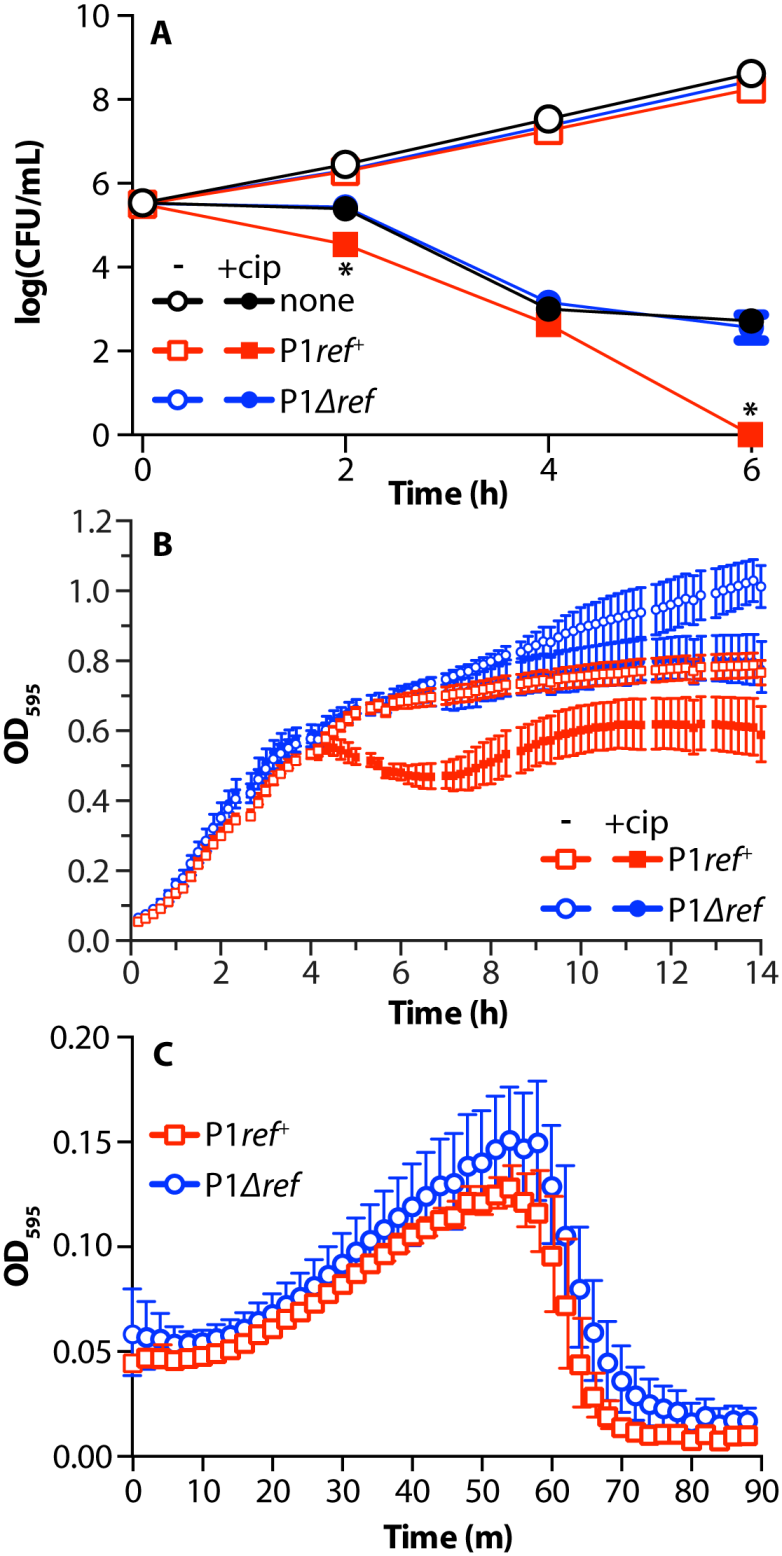
Phage-antibiotic synergy with bacteriophage P1 and ciprofloxacin. A. Survival of P1 lysogens after treatment with 8 ng/mL ciprofloxacin. Log phase cultures of *E*. *coli* MG1655, EAR2 (P1*ref+)* or EAW195 (P1*Δref*), were adjusted to 5x10^5^ CFU/mL and were treated with water (open markers) or 8 ng/mL ciprofloxacin (filled markers) with six hours outgrowth. The average CFU/mL and standard deviation of three biological replicates is reported (error bars are present, but smaller than markers in most cases). * = p-value<0.0001, when compared to MG1655 treated with 8 ng/mL ciprofloxacin. B. Optical density of P1 lysogens treated with ciprofloxacin. Log phase cultures of above strains were treated with water (open markers) or 4 ng/mL ciprofloxacin (filled markers) with outgrowth at 30°C. The average OD_595_ and standard deviation of five biological and two technical replicates for each condition is reported. C. Optical density of P1 lysogens induced with a temperature shift. Log phase cultures of strains in (B) were shifted from 30°C to 42°C to induce temperature-sensitive phage lysis and optical density was tracked as in (B). The average and standard deviation of three biological and three technical replicates is reported.

P1 lysogens having or lacking a functional *ref* gene, as well as non-lysogens were grown to log phase then treated with 8 ng/mL ciprofloxacin (Fig 1A). Immediately preceding treatment, and every two hours after for six hours, cells were removed from the culture, diluted, and plated. After an overnight incubation cells were counted to determine the number of viable *E*. *coli* cells at each post-infection time point. Non-lysogens, *ref*^*+*^ and *Δref* lysogens all grew similarly in the absence of ciprofloxacin (open markers). We next compared P1 lysogens and non-lysogens that were treated with ciprofloxacin, a DNA gyrase and topoisomerase IV inhibitor that creates double strand breaks in DNA by trapping covalent protein-DNA complexes during normal enzyme activity [55]. At 2 and 6 hours post-cipro treatment, there were significantly (p-value<0.0001) less WT P1 lysogens (*ref*^*+*^) that survived the ciprofloxacin treatment than non-lysogens. The killing enhancement observed in the P1 lysogens was entirely dependent on the presence of an intact *ref* gene. When the *ref* gene was deleted from the lysogen, survival after ciprofloxacin treatment returned to non-lysogen levels.

*E*. *coli* P1 lysogens’ response to ciprofloxacin treatment was also tracked by observing the optical density of the cultures over time in the presence and absence of a relatively low concentration of ciprofloxacin (4 ng/mL, 1/4–1/2x MIC) (Fig 1B). *Ref*^*+*^ P1 lysogens reached stationary phase at a lower cell density than *Δref* lysogens in the absence of ciprofloxacin treatment. When treated with ciprofloxacin, a portion of the P1 *ref*^*+*^ lysogens appeared to lyse upon reaching stationary phase, while *Δref* lysogens entered stationary phase normally. Treating *E*. *coli* cultures with 8 ng/mL ciprofloxacin for more than four hours in the presence of the *ref* gene led to complex filamentation and lysis phenotypes (as detailed later). By treating with 4 ng/mL ciprofloxacin, we were able to moderately reduce the effects of the antibiotic and view a less severe phenotype more suited to basic observation by optical density changes.

Entry into the lytic cycle was enhanced in *ref*^*+*^ lysogens only when the lytic cycle was induced using a DNA-damaging antibiotic. In the presence of a temperature shift, which cleaved the thermo-labile C1 repressor of P1Cm C1.100, the lysis profiles of both *ref*^*+*^ and *Δref* phage were identical (Fig 1C). Fig 1 demonstrates that the P1 *ref* gene is responsible for enhanced lethality of ciprofloxacin to *E*. *coli* P1 lysogens.

### The *ref* gene is a lytic function of bacteriophage P1

**Fig 2:**
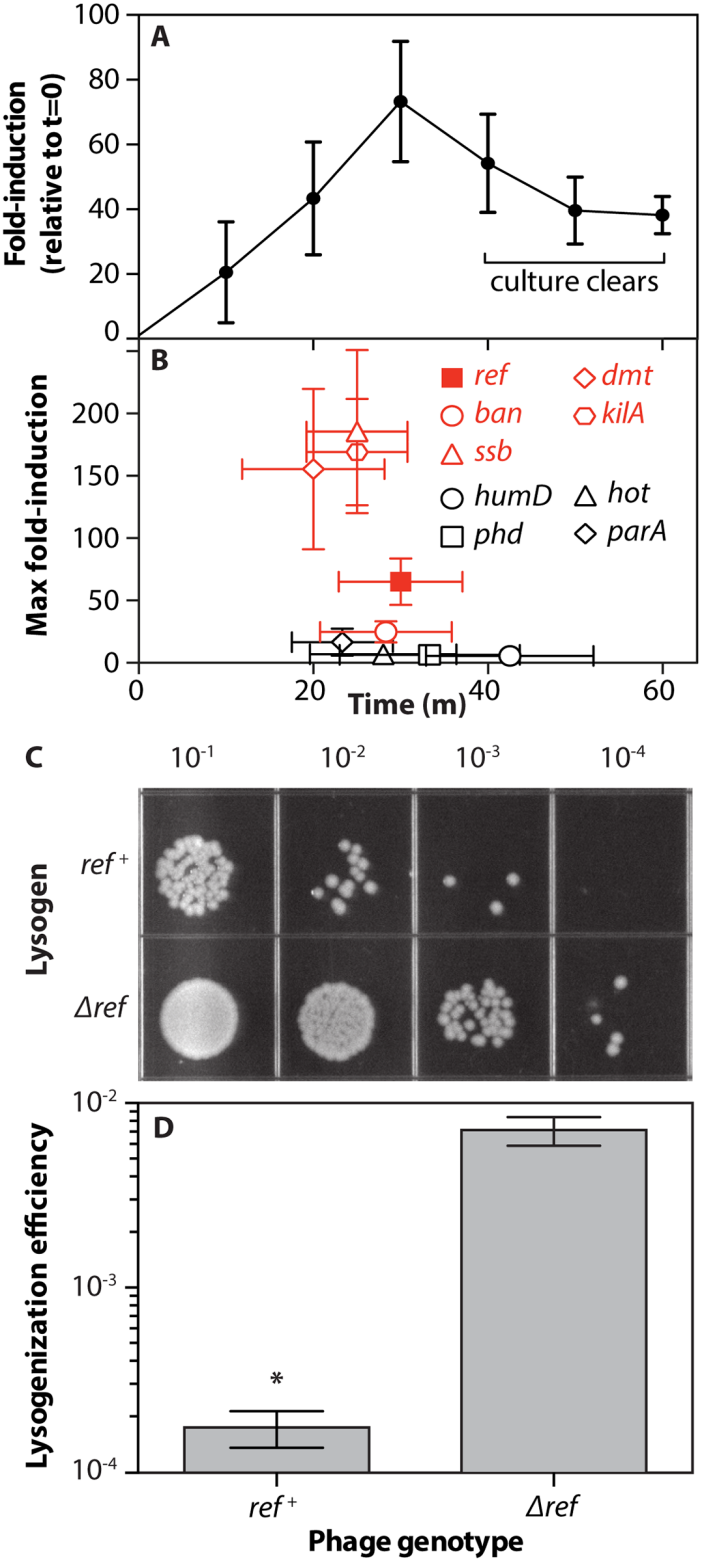
*ref* is a P1 lytic cycle gene. A. Rt-qPCR of P1 *ref* gene expression during temperature-induced lytic development of phage P1Cm C1.100. Total RNA was isolated and cDNA was obtained at times indicated. Portions of the *ref* (P1 phage) and *cysG* (*E*. *coli*) genes were amplified in triplicate using qPCR. Fold induction (y-axis) was calculated using the method of Livak and Schmittgen [56]. The average and standard deviation of three biological replicates is reported. B. Maximum fold-induction values for other P1 genes during the lytic switch, C1 repressor controlled genes = red. Fold-induction data was obtained and calculated as in A. Values within 20% of the maximum fold-induction for each gene in each biological replicate were averaged to obtain the maximum fold induction time and value for each gene. C. Lysogens formed upon infection of MG1655 with P1Cm C1.100. MG1655 culture was supplemented with CaCl_2_ and incubated with P1Cm C1.100 phage, followed by the addition of sodium citrate to chelate calcium ions and prevent superinfection. After recovery in LB, cells were serially diluted and plated on LB and LB/chloramphenicol (shown here) to select for phage lysogens. D. Colonies from plates in C were counted and the lysogenization efficiency (lysogens/viable cells) average and standard deviation from three biological replicate infections are reported. * = p-value<0.0001.

Next, we confirmed that *ref* gene expression occurs during the P1 lytic cycle. *E*. *coli* P1 lysogens (EAR2) harboring the thermo-labile phage C1 lytic repressor variant (C1.100) were grown to log phase at 30°C and then shifted to 42°C, inducing expression of all lytic cycle genes (Fig 2A and 2B). Levels of *ref* transcript were measured using rt-qPCR every 10 minutes following the temperature shift until cultures were clear, indicating completion of lysis between 40 and 60 minutes after lytic induction (Fig 2A). Expression of *ref* peaked at 30 minutes following lytic induction, with a 73-fold increase in expression, when normalized to levels measured prior to lytic induction. Changes in culture density during this period were controlled for by using exactly 1 μg of total RNA from each isolate as a template for reverse transcription. To put the fold-induction of the *ref* gene during lytic induction into context, the maximum fold-induction of four other P1 genes under the control of the C1 repressor and four P1 genes not under the control of the C1 repressor were determined using rt-qPCR on the same total RNA samples (Fig 2B). The maximum fold-induction of *ref* expression was higher than that observed for all of the genes not controlled by C1 repressor. It was also higher than the levels observed for *ban*, which is controlled by the C1 repressor. The expression of *ref* was lower and peaked slightly later after lytic induction than the C1 repressor-controlled genes *ssb*, *dmt*, and *kilA*. The induction of *ref* is thus within the range of induction seen for other C1 repressor-controlled genes during the lytic cycle.

Conversely, *ref* was shown to inhibit the establishment of *E*. *coli* P1 lysogeny (Fig 2C and 2D). Log-phase *E*. *coli* were incubated with phage containing or lacking the *ref* gene, then were diluted and plated on media selective for lysogens (Fig 2C) The presence of *ref* reduced the measured level of successful lysogenation by 40-fold. Thus, induction of the lytic cycle triggers the expression of *ref*. The presence of Ref protein natively can inhibit the establishment of lysogeny.

### *Ref* expression is repressed by *E*. *coli* H-NS

**Fig 3:**
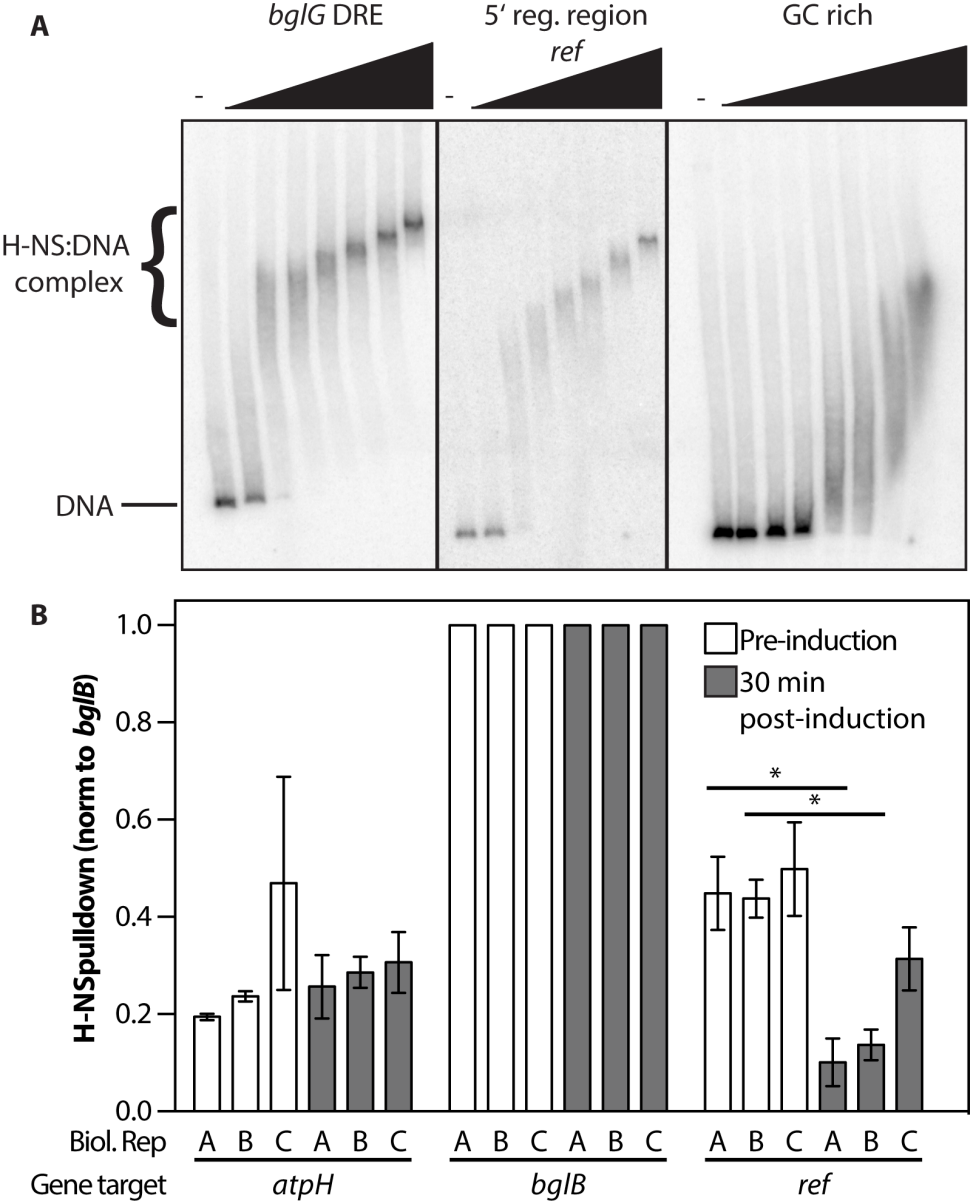
H-NS binds the 5' regulatory region of *ref*. A. Electrophoretic mobility shift assay (EMSA) demonstrating purified H-NS binding a radiolabeled DNA with sequence matching the 5' regulatory region of the *ref* gene. H-NS (0, 5, 10, 20, 50, 100, 200, and 500 nM) was incubated with radiolabeled DNA matching the high-affinity H-NS binding region (DRE) in *bglG*, *ref* gene 5' regulatory region, or a GC rich region from pBR322 (200 pM). DNA was run on a 4% polyacrylamide gel at 4°C, H-NS bound DNA runs slower than unbound DNA. Apparent K_d_ of H-NS binding to the *bglG* DRE or the *ref* 5’ regulatory region is 10 nM while the apparent K_d_ of H-NS binding to the GC rich region is 200 nM. *ref* samples were adjusted for intensity differences on the same gel for clarity. B. Digital droplet PCR (ddPCR) to quantify H-NS bound DNA pulled down during H-NS ChIP. Cells were grown to mid log phase, then lysis was induced using a temperature shift. Samples were harvested and proteins were crosslinked to DNA just before the temperature shift (lysogen) and 30 minutes later. An anti-H-NS antibody was used to ChIP H-NS-bound DNA. Concentrations of each gene target determined by ddPCR were normalized to positive control *bglB* DNA concentration and compared to the *atpH* gene which is considered a negative control for H-NS binding. *Ref* DNA concentration was normalized to the increased amount of phage DNA present due to lytic DNA replication. Each bar represents a single biological replicate amplified in technical quadruplicate with standard deviation. * = p-value<0.01

Given the apparent stress that *ref* expression places on the *E*. *coli* host, we thought it possible that the *ref* gene might be repressed not only by the phage C1 repressor, but also by at least one *E*. *coli* host factor. The 5′ regulatory region of *ref* contains a binding site for *E*. *coli* H-NS with sequence 5'-CGATAAA-3', a strong match to the H-NS consensus binding site 5'-tCGATAAATT-3' [57]. Purified H-NS bound the 5′ regulatory region of the *ref* gene, as demonstrated with an electrophoretic mobility shift assay (EMSA) using a radiolabeled dsDNA fragment with the sequence of the 5′ regulatory region of *ref* (Fig 3A). The affinity is similar to a documented high-affinity binding site of H-NS (the downstream regulatory element (DRE) of *bglG* [58]) and is stronger than the affinity for randomly selected GC-rich DNA, suggesting H-NS binds specifically to the 5′ regulatory region of *ref*.

H-NS is found in *E*. *coli* and other close relatives, such as *Salmonella typhimurium* [59]. It binds AT-rich DNA, which often includes virulence factors such as several of the pathogenicity islands in Salmonella [60, 61]. The *E*. *coli* genome has a GC content of 50.8%, while the P1 genome is somewhat more AT-rich, with a GC content of 47.3% [20]. H-NS binding nucleates at high-affinity binding sites [57, 62], leading to transcriptional silencing by interacting with RNA polymerase at promoter sequences (reviewed in [63]). In this way, *E*. *coli* can prevent the expression of horizontally acquired genes that may be detrimental to its survival, as tested here with *ref*.

In Fig 3B, binding of the 5′ regulatory region of *ref* in P1 lysogens is demonstrated using digital droplet PCR (ddPCR) and chromatin immunoprecipitation (ChIP). Lysis of EAR2 was induced with a temperature shift. Cells were cross-linked using formaldehyde just before the temperature shift and 30 minutes after the temperature shift. Using an anti-H-NS antibody, the ChIP assay pulled down *E*. *coli* and P1 DNA to which H-NS was bound. The presence and amount of specific genes in the IP sample were quantified using ddPCR. The actively transcribed gene *atpH* was used as a negative control for background levels of H-NS binding, whereas *bglB* is a positive control known to bind high levels of H-NS [64]. Signals for *ref* were normalized to input DNA at the 30 min time points to account for increases in the amount of P1 phage DNA compared to *E*. *coli* DNA during P1 lytic replication. The *ref* gene is bound by H-NS more strongly than *atpH* P1 lysogens, but the binding is significantly reduced 30 minutes after lysis is induced. Fig 3 demonstrates that H-NS is capable of binding the 5′ regulatory region of *ref*. This binding may result in added repression of *ref* in P1 lysogens, which is relieved during P1 lytic development.

### Expression of the N-terminal DNA binding domain of *Ref* triggers the *E*. *coli* SOS response

**Fig 4:**
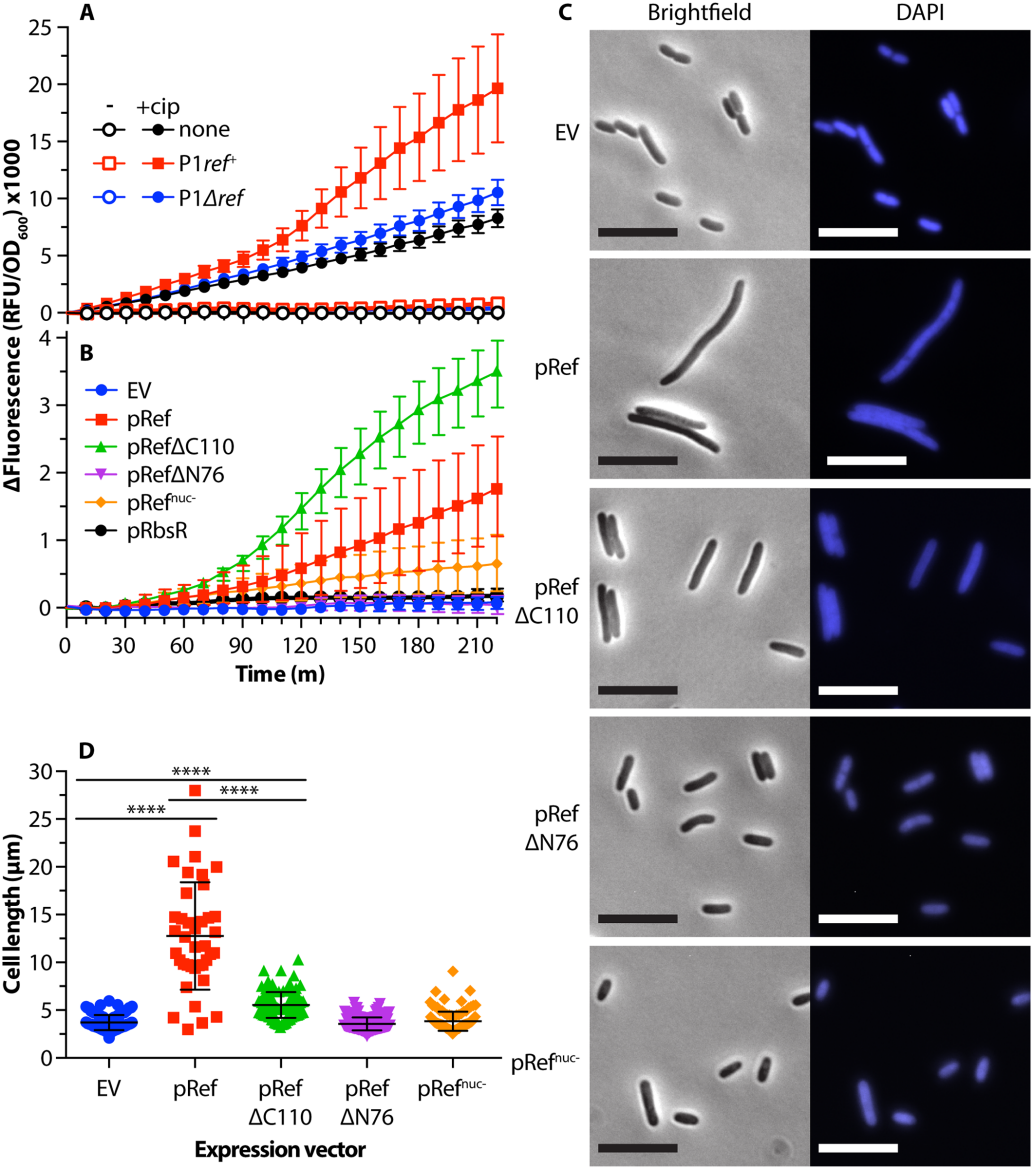
Expression of the N-terminal DNA binding domain of Ref induces the SOS response. A. Induction of the SOS response in P1 lysogens by 8 ng/mL ciprofloxacin. *E*. *coli* strains EAR15, EAR16, and EAR17 (non-lysogen, P1*ref*^*+*^lysogen, and P1*Δref* lysogen, respectively) were grown at 30°C. Each strain contained a plasmid (pEAW903) expressing SuperGlo GFP from the *recN* promoter, allowing the SOS response to be reported as fluorescence. After reaching log phase, ciprofloxacin (8 ng/mL) or water was added (time = 0) and growth continued under the same conditions. Δfluorescence (as calculated in Methods) average and standard deviation of three biological and four technical replicates is reported. B. SOS induction by strains expressing Ref in the absence of phage P1. *E*. *coli* strains EAR86 (EV), EAR87 (pRef) EAR88 (pRefΔC110), EAR 120 (pRefΔN76), EAR121 (pRef^nuc-^) and EAR123 (pRbsR) harboring protein expression plasmids and an SOS reporter plasmid were grown to log-phase at 30°C. Cultures were treated with 1% arabinose to induce protein expression (time = 0). Fluorescence and optical density were measured as in panel A. Normalized fluorescence average and standard deviation of at least two biological and two technical replicates is reported for each condition. C. Microscopy of *E*. *coli* cells expressing Ref variants. Log phase cultures of EAR61 (EV), EAR62 (pRef), EAR73 (pRefΔC110), EAR98 (pRefΔN76), and EAR105 (pRef^nuc-^) at 1x10^8^ CFU/mL were treated with 1% arabinose. Cells were outgrown for 4 hours and incubated with DAPI before imaging at 600x magnification using brightfield and fluorescence channels. Scale bar = 10 μm, representative images shown. D. Quantification of cell length data from (C) was obtained using the MicrobeTracker plugin for MatLab [66]. Each counted cell is represented by a single data point with the average and standard deviation for the data shown. At least 100 cells were measured for each condition, except WT Ref in which only 39 cells could be found. **** = p-value <0.0001.

We next turned to an exploration of the physiological effects of r*ef* gene expression. The endonuclease function of Ref [25] led us to hypothesize that *ref* expression might induce the bacterial SOS response. We used an SOS reporter plasmid encoding green fluorescent protein (GFP) under the early SOS-inducible *recN* promoter and transformed it into strains MG1655 (non-lysogen), EAR2 (P1*ref*^*+*^ lysogen) and EAW195 (P1*Δref* lysogen). Thus, the intensity and duration of the SOS response can be monitored by measuring cellular fluorescence at the emission maximum for GFP [65]. Once reaching log phase, cells were treated with ciprofloxacin at 8 ng/ml (1/2 the reported MIC for *E*. *coli* MG1655; see Methods). Growth was monitored by measuring optical density of the cultures at 600 nm and fluorescence was measured at 509 nm. Data was normalized as detailed in “Materials and Methods.” To account for reduced growth rates in some strains, fluorescence was divided by optical density. *Ref*^+^ lysogens show a higher level of expression from the early SOS *recN* promoter, when compared to a *Δref* lysogen or a non-lysogen (Fig 4A).

To confirm that the increased level of SOS seen in *ref*^*+*^ lysogens was due to *ref*, and not other phage functions or lysis of the cultures, a corollary experiment was carried out in non-lysogens harboring expression plasmids for Ref variants under the arabinose-inducible pBAD promoter (Fig 4B). In this and additional experiments, we also sought to evaluate the separate contributions of the unstructured N-terminal domain (Ref ΔC110 contains only the N-terminal 76 amino acid residues) and the C-terminal globular nuclease domain (Ref ΔN76). Another variant, Ref H134/153A (called Ref^nuc-^ below), retains both domains but lacks nuclease function due to replacement of two active site His residues that coordinate Zn ion.

Upon treatment with arabinose (to express proteins) in the absence of exogenous DNA damaging agents, cells expressing Ref variants that retained the N-terminal domain, including WT Ref, RefΔC110 (DNA binding domain only), or Ref^nuc-^, showed increased levels of SOS when compared to the empty vector control (EV). *E*. *coli* RbsR (a control for general effects of high levels of protein expression) or RefΔN76 (lacking the N-terminal DNA binding domain) produced SOS levels similar to the EV control. In the presence of arabinose and the exogenous DNA damaging agent ciprofloxacin, the effects were similar but more pronounced (S1 Fig). Normalized fluorescence/OD values cannot be directly compared between panels A and B because panel A utilized a high-copy reporter plasmid, while panel B utilized a low-copy plasmid to allow for a high copy Ref expression plasmid. These results indicated that Ref expression results in SOS induction. However, it was the highly charged and unstructured N-terminal DNA binding domain of Ref rather than the globular nuclease domain that was both necessary and sufficient to cause the SOS signal from the *recN* promoter.

### Ref expression produces SOS-independent cell filamentation

We also imaged cells studied in Fig 4B to determine if the cell filamentation that generally accompanies the SOS response was occurring as a result of Ref expression (Fig 4C). Cells were grown to 1x10^8^ CFU/mL and treated with 1% arabinose to express protein. As expected, the EV control and RefΔN76 expression did not produce filamentation, as they were not undergoing SOS. Cells expressing WT Ref protein and exhibiting high levels of SOS filamented extensively and were on average ~3x as long as EV control cells (quantified in Fig 4D). DNA was present throughout the length of the filaments, indicating that DNA replication was continuing even though cell divison was not. However, the N-terminal domain alone (RefΔC110) was insufficient to produce the same degree of cell filamentation, even though it was the construct most effective in the induction of SOS. Ref^nuc-^ expression did not generate cell filamentation, possibly due to SOS induction not reaching high enough levels. The RefΔC110 expression that generated the highest SOS levels produced a measurable increase in cell length when compared to EV control cells, but the filamentation was much reduced relative to the WT Ref expression. Overall, the results in Fig 4 demonstrate that the increased levels of the early SOS response seen in *E*. *coli* P1 lysogens are due almost entirely to the unstructured N-terminal DNA binding domain of Ref. While inducing SOS, this same N-terminal domain appears to suppress SOS-induced cell filamentation when it is separated from its native and active C-terminal nuclease domain. The entire protein is required for the highest levels of filamentation.

We further explored the effects of Ref expression on *E*. *coli* cell filamentation by expressing Ref in cells lacking or altering key host proteins involved in SOS-induced filamentation. As already noted, the RecA protein is required for SOS induction and is a cofactor for Ref-mediated DNA cleavage. SulA is an inhibitor of cell division [67, 68]. LexA3 is a non-cleavable variant of the LexA repressor of the SOS regulon [69, 70]. An absence of RecA or the presence of LexA3 should block SOS induction. The absence of SulA would normally suppress filamentation during SOS.

**Fig 5:**
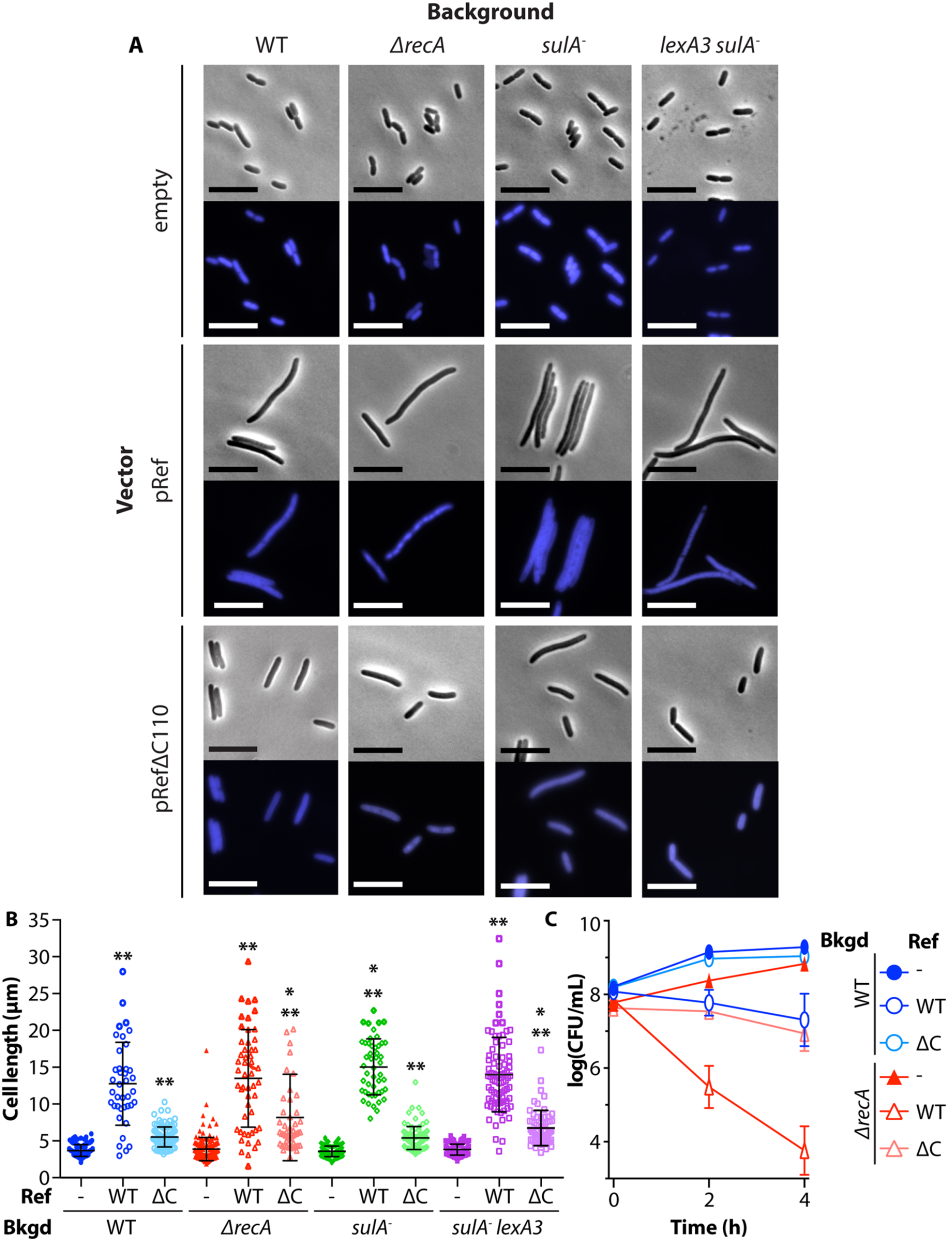
Ref expression causes cell filamentation independent of the *E*. *coli* SOS response. *A*. Microscopy of cells expressing Ref or RefΔC110. WT *E*. *coli* strains EAR61 (EV), EAR62 (pRef), EAR73 (pRefΔC110), *ΔrecA* strains EAR64 (EV), EAR65 (pRef), EAR74 (pRefΔC110), *sulA*^*-*^ strains EAR77 (EV), EAR78 (pRef), EAR79 (pRefΔC110), and *sulA*^*-*^ *lexA3* strains EAR69 (EV), EAR70 (pRef), and EAR75 (pRefΔC110) were grown to 1x10^8^ CFU/mL, treated with 1% arabinose, outgrown for 4 hours, and images were obtained as in Fig 4C. Scale bar = 10 μm, representative images shown. B. Quantification of cell length data from (A) was obtained using the MicrobeTracker plugin for MatLab. Each counted cell is represented by a single data point with the average and standard deviation for the data shown. An average of 90 cells (range: 39–188) were counted for each condition. ** = p-value <0.0001 when compared to EV in same background, * = p-value <0.05 when compared to same vector in WT background. C. Cell survival after expression of Ref. WT *E*. *coli* strains EAR61 (EV), EAR62 (pRef), EAR73 (pRefΔC110), *ΔrecA* strains EAR64 (EV), EAR65 (pRef), EAR74 (pRefΔC110) were treated as in (A). Cells were plated for viability and the average and standard deviation of at least three biological replicates for each condition are reported (error bars small and not visible in some cases). Significant p-values are noted in S1 Table.

Plasmids encoding the EV control, WT Ref and RefΔC110 expression were introduced into *ΔrecA*, *sulA*^*-*^, and *lexA3 sulA*^*-*^ backgrounds. Log-phase cells (1x10^8^ CFU/mL) were treated with arabinose to induce protein expression, allowed to continue growing for four hours, stained with DAPI, and imaged using brightfield and fluorescent illumination (Fig 5A). Cells expressing WT Ref and RefΔC110 (the N-terminal domain alone) showed a statistically significant increase in cell length when compared to the EV control in all four backgrounds tested. However, expression of WT Ref protein always generated much longer cells than RefΔC110 expression (quantified in Fig 5B, ** = p-value<0.0001). WT Ref expression produced the same level of filamentation in all backgrounds, while RefΔC110 expression produced somewhat longer cells in *ΔrecA* and *sulA*^*-*^*lexA3* backgrounds than in WT or *sulA*^*-*^ backgrounds (p-value<0.05 indicated with *). In all filamentous cells, DNA was present throughout the length of the filament, indicating that DNA replication continued in all genetic backgrounds even though cell division was compromised. Expression of Ref protein causes filamentation through an undefined pathway that is clearly independent of the SOS-induced, SulA-dependent pathway.

In Fig 5C, survival of the WT and *ΔrecA* cells in Fig 5A and 5B were tracked by diluting and plating just before arabinose addition to induce expression of Ref or Ref variants (t = 0 hours), and every 2 hours thereafter. Expression of WT Ref causes cell death in both WT and *ΔrecA* backgrounds, with *ΔrecA* cells showing a 4-log reduction in viability four hours after Ref induction. Expression of RefΔC110 has no effect on cell viability in a WT background, but reduces cell viability in a *ΔrecA* background in a manner comparable to WT Ref expression in a WT background. Fig 5C demonstrates that Ref expression is toxic when induced in late log-phase cells, and that this occurs to some degree independently of *recA*. Ref-mediated lethality is thus not uniquely a function of the RecA-dependent nuclease activity of Ref.

### Ref expression is lethal to *E*. *coli* cells entering stationary phase, and can improve the effectiveness of DNA-damaging antibiotics

As shown in Fig 1B, P1*ref*^*+*^ lysogens reach stationary phase at lower cell densities, and Fig 5C demonstrates that Ref expression is toxic to cells treated in late log-phase even in the absence of ciprofloxacin. A major problem in the treatment of infections of bacterial pathogens is the generation of persister cells that survive treatment and lead to chronic infection. The persistence is often tied to the bacterial SOS response and/or slow growing (stationary phase) bacteria [47].

**Fig 6:**
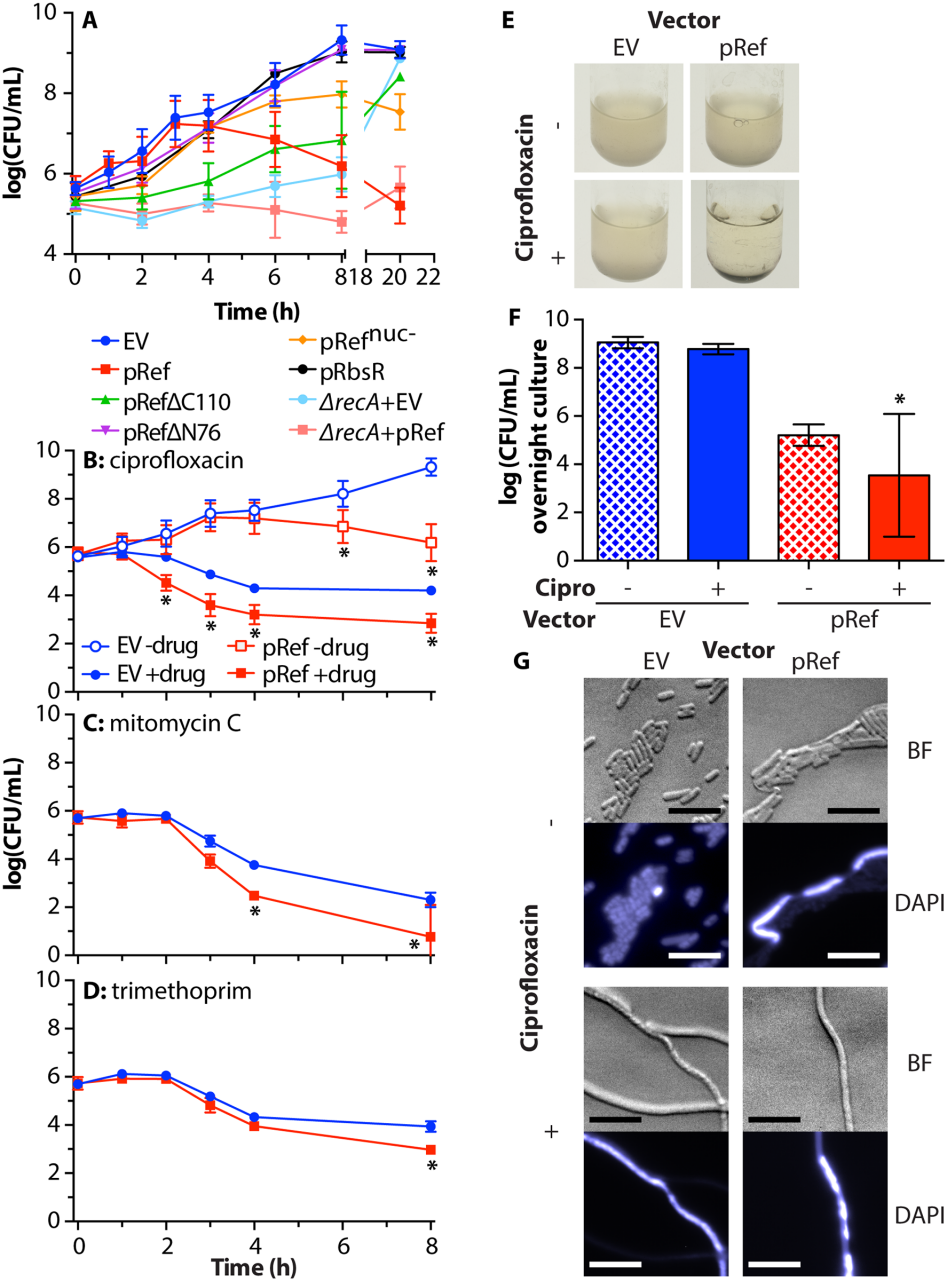
Ref is toxic to *E*. *coli* in stationary phase and enhances lethal effects of DNA-damaging antibiotics. A. Cell survival during Ref and variant expression. WT *E*. *coli* strains EAR61 (EV), EAR62 (pRef), EAR73 (pRefΔC110), EAR98 (pRefΔN76), EAR105 (pRef^nuc-^), EAR104 (pRbsR), and *ΔrecA* strains EAR64 (EV) and EAR65 (pRef) were grown to log-phase, adjusted to 5x10^5^ CFU/mL, treated with 1% arabinose, and outgrown for one day at 30°C. Cells were plated and the average and standard deviation of CFU/mL for at least three biological replicates for each condition are reported. Significant p-values can be found in S1 Table. B. WT *E*. *coli* strains EAR61 (EV) and EAR62 (pRef) were grown as described in (A), but with the addition of water or 8 ng/mL ciprofloxacin in addition to 1% arabinose. Cells were plated and data was reported as in (A). * = p-value<0.001, when compared to EV with same treatment. C. Same as (B) except 5 μg/mL mitomycin C was used instead of ciprofloxacin. * = p-value<0.001, when compared to EV with same treatment. D. Same as (B) except 0.5 μg/mL trimethoprim was used instead of ciprofloxacin. * = p-value<0.001, when compared to EV with same treatment. E. Images of cultures from (B) after overnight incubation. F. Quantification of viable cells in panel (E). Average and standard deviation of 4–8 biological replicate cultures are reported. * = p-value<0.001, when compared to EV with same treatment. G. Microscopy of cells in panel (E), obtained as in Fig 4C. Scale bar = 10 μm.

To further explore the molecular basis of Ref toxicity, we explored the effects of Ref over longer time periods. We treated *E*. *coli* in early log-phase (5x10^5^ CFU/mL) with arabinose to express Ref variants. As cells grew, any inhibition of entry into stationary phase could be observed by diluting and plating the cultures periodically (Fig 6A). Cells expressing WT Ref (red squares) grew normally for approximately 4 hours, but began to die rapidly after 4 hours, and cells did not recover overnight (compare to EV control, blue circles). Thus, Ref has little effect in early log phase, but toxicity becomes apparent as cells approach stationary phase. Expressing Ref^nuc-^ resulted in a similar, although substantially less dramatic phenotype, indicating that Ref nuclease activity is at least partially responsible for cell death. When the DNA binding domain of Ref was removed (RefΔN76, purple triangles), cells grew normally, indicating that Ref DNA binding is required for toxicity. Expression of the Ref DNA binding domain only (RefΔC110, green triangles) showed a slow growth phenotype, but cells eventually recovered from treatment overnight. The N-terminal DNA binding domain of Ref thus slowed cell division, probably by slowing replication and/or inducing the SOS response. Long-term toxicity was minimal. This result indicates that the Ref nuclease domain is also required for toxicity. Additionally, the *E*. *coli* protein RbsR was under the same promoter and conditions (black circles) as a control, and had no effect on cell growth or viability, indicating that cell death was not a general effect of high level protein expression. In a *ΔrecA* background, cells expressing Ref showed minimal growth or death, diverging from EV control cells between 6 and 8 hours after Ref induction and (unlike the EV control) never approached stationary phase.

The effect of expressing Ref in combination with various antibiotics was then examined (Fig 6B–6D). Cultures of WT *E*. *coli* were grown to log phase as above and treated with 1% arabinose and various antibiotics (-drug data in Fig 6B is also shown in Fig 6A). Ciprofloxacin (Fig 6B) and mitomycin C (Fig 6C) both function as antibiotics by creating DNA damage [71, 72], and both are capable of inducing the SOS response [53, 73]. Ref expression enhanced the antibiotic effectiveness of both of these antibiotics, decreasing survival by approximately 2-logs (red squares) compared to the EV controls (blue circles). Trimethoprim functions by inhibiting bacterial dihydrofolate reductase, a key enzyme in producing thymidine for DNA replication [74]. Although trimethoprim can somewhat induce the SOS response [75], it does so indirectly, as it does not produce DSBs *de novo*. *E*. *coli* sensitivity to trimethoprim was only moderately increased (less than 1 log) by the expression of Ref (Fig 6D).

Ref reduces the capacity of *E*. *coli* to recover from ciprofloxacin treatment. Strains from Fig 6B were allowed to continue growing overnight after treatment with arabinose with or without ciprofloxacin. Images of the cultures are shown in Fig 6E, with corresponding viability counts in Fig 6F and brightfield and DAPI-stained fluorescent microscopy images in Fig 6G. Cells harboring the EV control in the absence of ciprofloxacin continued to grow and maintain normal viability and cell morphology (Figs 6E–6G). In the presence of ciprofloxacin, these same cells recovered viability overnight, although a large number of dead cells are seen in the microscopy images (indicated by cells where DAPI fluorescence saturated the camera, as dead cells are more permeable to DAPI.). Cells expressing Ref in the absence of ciprofloxacin did not recover viability overnight (approx. 4 log reduction in viability, compared to EV control, Fig 6F), are filamentous, and large numbers of dead cells with high DAPI permeability are seen (Fig 6G). Dead cells are not seen in Figs 4C and 5A, because Ref had only been expressed for 4 hours at the time the images were recorded. In the presence of ciprofloxacin, cells expressing Ref exhibited a range of viability phenotypes in seven trials after overnight incubation, from numbers resembling Ref expression in the absence of ciprofloxacin (4 log reduction, compared to EV control) to a completely non-viable culture with no recoverable colonies (9 log reduction; two of seven trials). Thus, under these conditions, Ref expression can complement ciprofloxacin treatment as it reduces the capacity of *E*. *coli* cells to recover from ciprofloxacin treatment.

**Fig 7:**
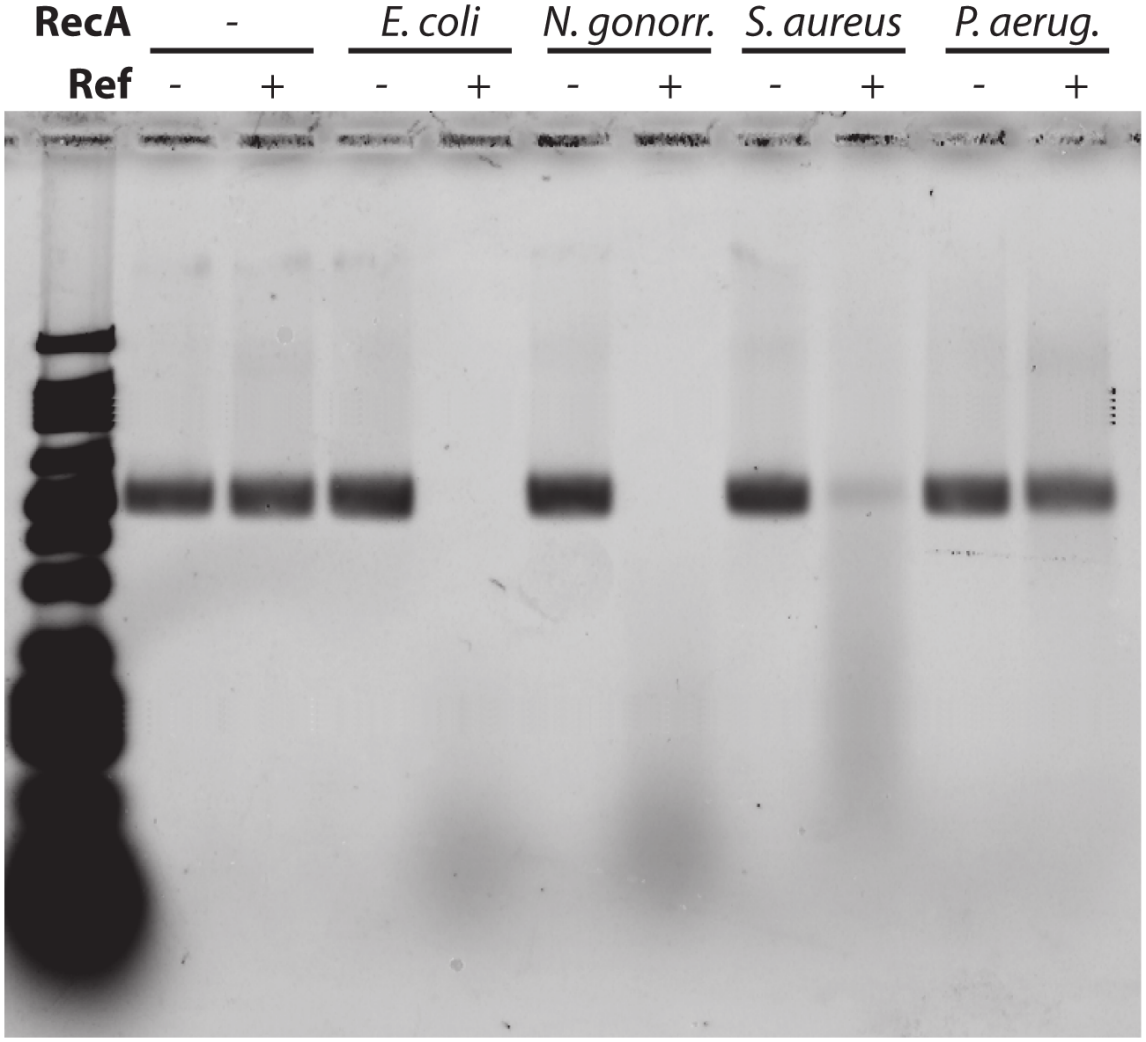
Ref protein can cleave DNA bound by RecA from pathogenic bacterial species. Agarose gel of circular single-stranded (css) DNA (4 μMnt) incubated with RecA protein from different bacterial species (2.4 μM) and Ref (100 nM) for 20 minutes at 37°C. All cssDNA bound by *E*. *coli* and *N*. *gonorrhoeae* RecA protein was degraded within 1 hour. P1 Ref protein showed slightly reduced nuclease activity on DNA bound by RecA from *Staphylococcus aureus* and minimal low activity on DNA bound by RecA from *Pseudomonas aeruginosa*.

In principle, Ref should produce toxicity in any bacterial species with a suitable RecA protein that can act as a partner in Ref-mediated genomic DNA cleavage. To provide a proof-of-concept that Ref could augment antibiotic treatment in other bacterial species, purified Ref protein was incubated with single-stranded DNA (ssDNA) coated with purified RecA homologs from four bacterial species: *Escherichia coli*, *Neisseria gonnorhoeae*, *Staphylococcus aureus* and *Pseudomonas aeruginosa* (Fig 7). Ref degrades ssDNA bound by the *E*. *coli* RecA filament, as observed previously (lane 4) [25]. Ref will also degrade ssDNA bound by the *N*. *gonnorhoeae* RecA protein and the *S*. *aureus* RecA protein. Minimal cleavage is seen with the *P*. *aeruginosa* RecA filament. The results in Fig 7 indicate that Ref could, in principle, create DNA damage in pathogenic bacterial species by cleaving RecA filaments within the bacterial cells.

## Discussion

There are three major conclusions to this work. First, *ref* is a phage P1 lytic cycle gene. Its physiological function is to enhance lethality when the P1 lytic cycle is induced by DNA damage. Both the DNA-binding and nuclease activity of Ref contribute to this phenotype. Second, Ref inhibits cell division independent of SOS-mediated inhibition of cell division, and this effect is partially dependent on the C-terminal nuclease domain of Ref. Finally, a substantial enhancement of antibiotic lethality is observed when an *E*. *coli* P1 lysogen is treated with DNA damaging antibiotics. The *ref* gene is both necessary and sufficient to account for the P1 phage contribution to bacterial lethality. The effect is reprised by expressing Ref protein in the absence of other phage functions. The DNA-binding domain of Ref is required for this function, and Ref nuclease activity contributes.

The effects of Ref are amplified when the protein is expressed in the absence of other phage functions. Ref toxicity increases as cells approach stationary phase. The highly charged N-terminal DNA binding domain of Ref, and the C-terminal globular nuclease domain, both contribute to the observed effects. The N-terminal domain represents a novel DNA-binding motif, inherently unstructured and with 25 of 76 amino acid residues positively charged at neutral pH [26]. Expression of these 76 amino acids on their own (Ref ΔC110) results in induction of the SOS response (Fig 4) and decreased growth rates (Fig 6). However, cells recover from Ref ΔC110 expression alone if given sufficient time. The C-terminal domain (RefΔN76) has little effect on its own. Expression of the complete Ref protein confers considerable toxicity in *E*. *coli*, especially when expressed in the presence of moderate doses of ciprofloxacin. Expression of Ref results in substantial cell filamentation even in genetic backgrounds that block SOS induction (Fig 5). As the filamentation occurs even in the absence of RecA, we hypothesize that Ref may inhibit cell division via a mechanism unrelated to its nuclease function.

**Fig 8:**
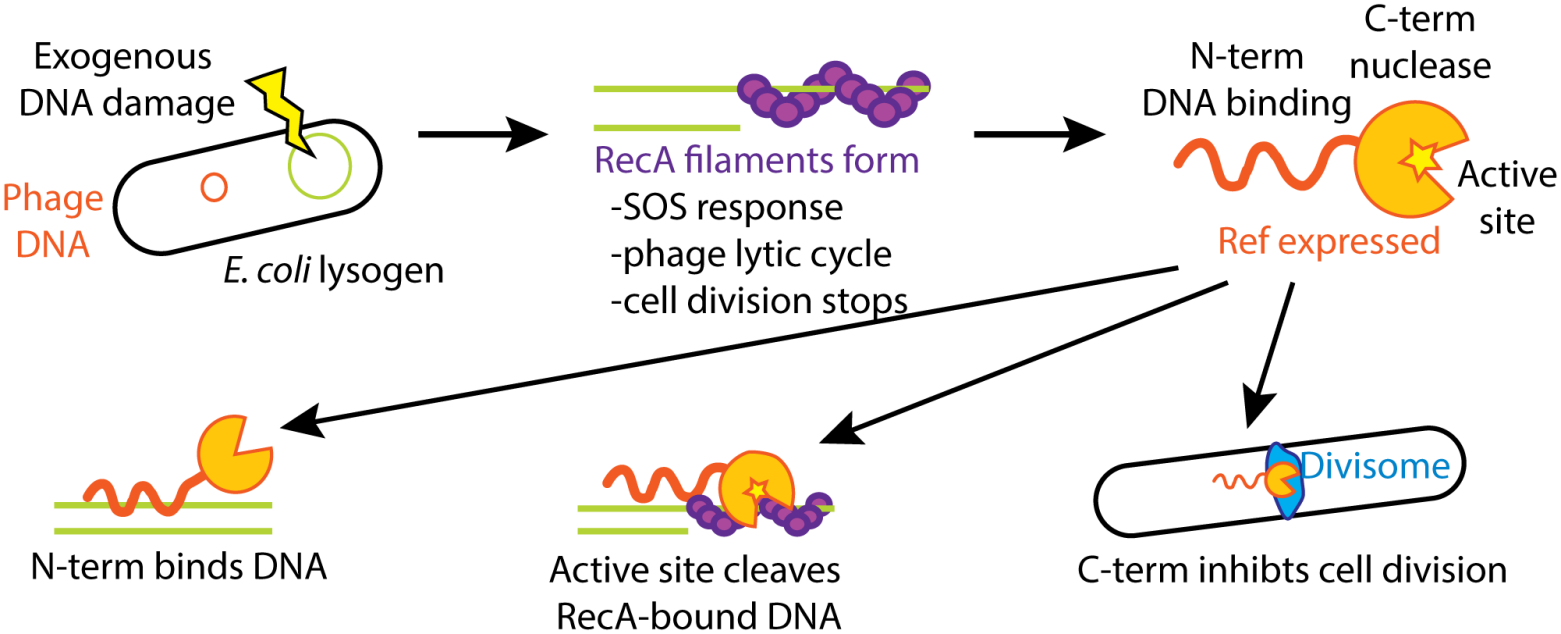
Multiple roles for Ref protein during the P1 lytic cycle. When a P1 lysogen is exposed to a DNA damaging agent (such as cipro), RecA filaments form near the damaged DNA sites. This causes (1) *E*. *coli* SOS response induction, (2) induction of phage lytic cycle, (3) inhibition of cell division. Ref protein is produced during the phage lytic cycle and performs three functions: (1) the Ref N-terminal DNA binding domain binds bacterial DNA, inhibiting other DNA metabolism enzymes, (2) the nuclease active site of Ref cleaves RecA-bound DNA to enhance the bacterial SOS response, (3) Ref inhibits cell division mainly *via* its C-terminal domain, and possibly through an interaction with the bacterial divisome.

We propose a model of Ref action during the bacteriophage P1 lytic cycle, in which Ref converts the *E*. *coli* SOS response to a bacterial liability. The strategy is employed when DNA damage occurs and the host cell (and thus the lysogenic phage) is in jeopardy (Fig 8). When *E*. *coli* lysogens are subjected to DNA damage, RecA filaments form on exposed single-stranded DNA. This triggers the induction of the bacterial SOS response, which in turn will block cell division and induce the phage lytic cycle [76–79]. Ref is expressed as part of the P1 lytic cycle and acts to enhance the lytic cycle in several ways. First, Ref can bind DNA through its N-terminal domain, creating blocks to other DNA metabolism enzymes that must access the DNA. Second, Ref can cleave DNA bound by RecA filaments, which are continually present during the SOS response. The creation of additional DNA damage sets the cell into an amplification cycle of more RecA filaments forming, the lytic cycle of phage replication continuing, and Ref expression leading to additional DNA damage. Finally, Ref can inhibit cell division independently of the SOS response through a yet-undiscovered mechanism. How each of these functions could benefit the phage is explored in more detail below.

Ref expression occurred during the lytic cycle (Fig 2A), as expected due to the presence of a C1 repressor binding site occluding one of the *ref* promoters [20]. The *ref* induction levels during the lytic cycle fell within a range seen from other genes with C1 repressor binding sites (Fig 2B). Conversely, the presence of *ref* inhibited the lysogenic cycle of the phage (Fig 2C and 2D). These results, combined with previous research showing that *ref* activity from its native promoter was noticeably higher in cells with defective or absent C1 repressors [16, 17], firmly establishes ref as a lytic cycle gene of P1. *E*. *coli* lysogens benefit strongly from the repression of lytic phage genes, so it was not surprising that the *E*. *coli* xenogenic silencer H-NS also bound to the 5′ regulatory region of *ref*, perhaps complementing C1 in repressing *ref* expression.

The SOS response plays a key role in the biological rationale for the evolution of the Ref endonuclease. The presence of the *ref* gene, even in the absence of other phage functions, enhances the *E*. *coli* SOS response (Fig 4A and 4B). Quinolone antibiotics, like ciprofloxacin, also induce the SOS response [71]. If *ref* is deleted from the P1 chromosome, the SOS response in *E*. *coli* P1 lysogens returns almost to non-lysogen levels. The DNA-binding domain of Ref is required for SOS induction in the absence of a DNA-damaging antibiotic, as expression of RefΔN76 results in no detectable SOS response. Ref nuclease activity is at least partially responsible for SOS enhancement, as expression of the active site variant Ref H134/153A reduces the SOS response to about half the levels seen with WT Ref. However, deleting the entire nuclease domain (RefΔC110) results in an even higher SOS response than caused by WT Ref expression. This somewhat surprising result may be due to the elimination of interaction sites for other proteins on the C-terminal globular domain, freeing up all available Ref N-terminal domains to bind DNA.

There are several possible ways the phage can benefit from ensuring that the host cell remains in SOS. First, a binding site for the SOS repressor LexA occurs in the bacteriophage P1 genome, overlapping the promoter for P1 *coi* [20]. During SOS, the Coi protein is expressed and binds to P1 C1 repressor, preventing it from repressing lytic cycle genes [24]. Lytic replication is the only way for a phage to survive if the host is unable to repair DNA damage via the SOS response, making this coupling of SOS and lytic replication crucial for phage survival. Second, the main subunit of *E*. *coli* DNA polymerase III (*dnaE*) is essential for the lytic replication of bacteriophage P1 [80]. SOS promotes the use of the Y-family error-prone polymerases Pol IV and Pol V for host replication at sites of damage, freeing up Pol III for phage replication [81–83].

The DNA binding activity of Ref is centered in the unstructured N-terminal domain [26]. Ref binding of DNA appears to play a role in all phenotypes we observe with Ref expression, as RefΔN76 does not induce SOS, cause cell filamentation, or lead to cell death (Figs 4B, 4C and 6A). DNA binding proteins can stall replication forks, creating gaps and breaks in the DNA [84]. Most replisome stalling is due to interactions with DNA-protein complexes [85]. Replication restart pathways are needed, and if the fork is not reloaded DNA damage will persist [86].

Actively transcribing RNA polymerase complexes must also contend with DNA-bound proteins and generally pause when encountering other DNA-protein complexes [87]. Finally, Ref-DNA complexes present a barrier to RecA-mediated DNA repair, a function required for bacterial survival in the presence of DNA-damaging agents. DNA binding activity could explain why Ref is still toxic in the absence of *recA*, even though RecA is required to create a substrate for Ref endonuclease activity [25].

The phage genome would not be immune from the DNA binding effects of Ref, but the presence of many copies of the phage genome during the lytic cycle would mitigate any effects. A single instance of Ref binding to the *E*. *coli* genome that results in unrepaired DNA damage will lead to death of the host. To contrast, a single instance of Ref binding to the phage genome that results in unrepaired DNA damage will reduce the burst size by a single phage.

Ref cleaves RecA-bound DNA *in vitro* [25–27]. It is likely that Ref’s RecA-dependent DNA cleavage activities elevated DNA damage in P1-infected cells in the original studies on Ref [16–18, 29]. Ref only affected lysis of P1-infected cells in the presence of DNA damage, and had no effect when lysis was induced using the temperature sensitive C1 repressor (Fig 1B and 1C). This indicates that in P1 lysogens, DNA damage is required for the cellular toxicity of the Ref protein, and is in line with our hypothesis that Ref creates additional DNA damage by cleaving DNA within RecA filaments in a P1 lysogen. This may explain the general lack of a *ref* phenotype observed in the earlier studies [16–18, 29]. Ref’s nuclease activity is at least partially responsible for the cellular toxicity of Ref, as expression of the active site variant H134/153A results in about half the SOS response and toxicity as expression of WT Ref (Figs 4B and 6A).

Finally, Ref inhibits cell division independently of the SOS response. There are several examples of phage-encoded proteins that have this same phenotype: λ Kil and T7 gene product 0.4, both of which directly inhibit the assembly of bacterial FtsZ [88, 89]. While one might expect a nuclease like Ref to inhibit cell division by cleaving the DNA being segregated to daughter cells, the filamentation observed here was independent of nuclease activity (Fig 4C and 4D) and the SOS response (Fig 5). We are currently investigating potential interactions between Ref and the divisome in more detail. At this time, it is unclear exactly what benefit delaying cell division confers to the phage. Others have suggested that phage inhibition of cell division will increase host adaptations to stress [90] or prevent compartmentalization of the host that would interfere with phage replication or packaging [88]. Either explanation could fit our data.

Ciprofloxacin treatment of *E*. *coli* lysogens may be an example of phage-antibiotic synergy (PAS) [10], with our data supporting a model not yet seen in other cases of PAS. Most antibiotics are effective only if cells are in active metabolism, and bacteria can persist through antibiotic treatment if they enter stationary phase [47]. Ref expression is clearly most toxic as cells approach stationary phase (Fig 6A). Thus, the toxicity of Ref complements the toxicity of most antibiotics. Ref nuclease activity and DNA binding contribute to this activity. Since the identity of Ref as an HNH endonuclease family member was not originally evident from its gene sequence [25], it is tempting to speculate that other families of phage-encoded endonucleases with a function similar to Ref may exist.

There is potential for more general use of Ref in therapeutic applications of phage. The use of toxic phage proteins of various classes as alternatives or complements to antibiotics has been examined previously, but Ref is not a member of any of these classes [91]. Ref will utilize RecA proteins from at least two prominent pathogens (*Neisseria gonnorhoeae* and *Staphylococcus aureus*) as cleavage partners. Since no other P1 functions are required for its synergistic effect with antibiotics, the *ref* gene is portable. In principle, *ref* could be cloned into any of a wide range of phages that are being tested for use in agriculture and medicine. We propose that any antibiotic that induces the bacterial SOS response may in principle be rendered more effective in the presence of a complementing phage expressing the *ref* gene as a cloned adjuvant.

## Materials and Methods

### Reagents/media/growth conditions

For liquid growth, cells were grown in Luria-Bertani (LB) broth at 37°C with 250 rpm shaking, unless otherwise noted. P1 lysogens were always grown at 30°C to prevent the induction of the thermo-inducible CI repressor, which would cause phage to enter the lytic cycle. For growth on solid medium, cells were plated on LB plus 1.5% (wt/vol) agar. M9 minimal media was prepared using a standard recipe [92]. Chloramphenicol (MP Biomedicals) was dissolved in 95% ethanol to make a 25 mg/mL stock solution and was used at 25 μg/mL for all selections. Ampicillin (Sigma) was dissolved in water to make a 100 mg/mL stock and was used at 100 μg/mL for all selections. Ciprofloxacin was dissolved in water at 5 mg/mL. Mitomycin C was dissolved in water at 0.5 mg/mL and stored protected form light. Trimethoprim was dissolved in DMSO at 5 mg/mL. All antibiotics were serially diluted in water to obtain working concentrations. L-arabinose was dissolved in water at 20% (w/v) and used at a working concentration of 1% (w/v) to induce protein expression from pBAD-myc-HisA-derived plasmids.

### Preparation of phage from lysogens

Phage stocks (listed in Table 1) were prepared from *E*. *coli* lysogens using a modified plate lysis method. Overnight cultures of lysogen (100 μL) were mixed with 3 mL melted and cooled top agarose (LB+0.7% agarose), and 15 mM CaCl_2_ and poured on top of a room-temperature LB plate. The plate was incubated right-side up at 42°C overnight to allow phage to lyse the cells. The next day, 5 mL SM buffer (5.8 g NaCl, 2 g MgSO_4_ 7 H_2_O, 50 mL 1 M Tris-Cl pH 7.5, 5 mL 2% gelatin in 1 L H_2_O) was added to each plate and rocked at 4°C for 2 hours. The SM buffer (with phage) was transferred to a sterile glass test tube and a few drops of chloroform were added to kill any remaining *E*. *coli*. The tube was spun down to pellet cell debris, and the supernatant was removed and stored in a fresh, sterile glass test tube at 4°C. Phages were titered using standard methods [92] to ensure similar PFU/mL (within 1 log) were obtained.

**Table 1:** Strains, phage, and plasmids used in this study.

Strain, Phage, Plasmid	Relevent Characteristics	Reference or Source
Strain		
BR4666	P1Cm C1.100 lysogen	M. Yarmolinsky
EAR2	MG1655, P1Cm C1.100 lysogen	This paper
EAW195	MG1655, P1Cm C1.100*Δref*::*Kan* lysogen	“
EAW13	MG1655*sulA-*	[93]
EAW20	MG1655*ΔrecA*	This paper
EAW28	MG1655*sulA-lexA3*	“
EAR15	MG1655 pEAW903	“
EAR16	EAR2 pEAW903	“
EAR17	EAW195 pEAW903	“
EAR61	MG1655 pBAD-myc-HisA	“
EAR62	MG1655 pEAW974	“
EAR64	EAW20 pBAD-myc-HisA	“
EAR65	EAW20 pEAW974	“
EAR69	EAW28 pBAD-myc-HisA	“
EAR70	EAW28 pEAW974	“
EAR73	MG1655 pEAR12	“
EAR74	EAW20 pEAR12	“
EAR75	EAW28 pEAR12	“
EAR77	EAW13 pBAD-myc-HisA	“
EAR78	EAW13 pEAW974	“
EAR79	EAW13 pEAR12	“
EAR86	MG1655 pEAW915 pBAD-myc-HisA	“
EAR87	MG1655 pEAW915 pEAW974	“
EAR88	MG1655 pEAW915 pEAR12	“
EAR98	MG1655 pEAW1033	“
EAR104	MG1655 pEAW427	“
EAR105	MG1655 pEAR21	“
EAR120	MG1655 pEAW915 pEAW1033	“
EAR121	MG1655 pEAW915 pEAR21	“
EAR123	MG1655 pEAW915 pEAW427	“
Phage		
P1Cm C1.100	Thermoinducible P1	M. Yarmolinsky
P1Cm C1.100*Δref*::*Kan*	Thermoinducible P1 *Δref*	This paper
Plasmid		
pBAD-myc-HisA	Empty vector for expression from pBAD promoter experiments	Invitrogen
pBR322	Template for GC EMSA	New England Biolabs
pEAR12	RefΔC110 expression from pBAD promoter	This paper
pEAR21	Ref H134/153A expression from pBAD promoter	“
pEAW413	Used to delete *recA* from MG1655 chromosome	“
pEAW427	RbsR expression from pBAD promoter	“
pEAW507	For replacing genes on chromosome with KanR cassette	[94]
pEAW584	WT Ref expression from T7 promoter	[25]
pEAW685	Ref ΔN76 expression from T7 promoter	“
pEAW903	*precN-gfp* (AmpR) for use as SOS reporter	This paper
pEAW974	Ref expression from pBAD promoter	“
pEAW915	*precN-gfp* (CmR) for use as an SOS reporter	[94]
pEAW1033	RefΔN76 expression from pBAD promoter	This paper
pKD46	Encodes λred recombinase genes	[95]
pMK110	Template for *bglB* EMSA	[96]

### Plasmid construction

Plasmid pEAW903 is Super-Glo GFP under the control of the *E*.*coli recN* promoter. The 200 bp upstream of the *E*. *coli recN* gene were inserted into pQBI63 (Qbiogene), replacing the T7 promoter. PCR was used to generate the 200bp upstream of *recN* gene with *E*. *coli* MG1655 DNA as template, and primers consisting of a BglII site followed by MG1655 bases 2749617–2749637, and a NheI site followed by a NdeI site (bases 5326–5315 of pQBI63) and bases 2749813–2749793 of MG1655. The PCR product was digested with BglII and NheI and ligated into pQBI63 digested with the same enzymes. Plasmid pEAW915 was functionally similar, but contained the *recN* promoter fused to Super-Glo GFP in the pACYC184 background, allowing for plasmid selection with chloramphenicol [94].

Plasmid pBAD-myc-His A was purchased from Invitrogen and used as an empty vector control for all experiments where Ref was expressed. To construct pEAW974 (WT Ref in pBAD), pEAW584 (WT Ref in pET21A) [25] was used as a template in a PCR with an upstream primer consisting of a BspHI site, followed by bases 5–26 of the *ref* gene. The ACA coding for Thr at aa 3 was changed to ACC for better codon use. The downstream primer consisted of a BamHI site, followed by the last 18 bases of the *ref* gene. The PCR product was digested with BspHI and BamHI, and inserted into pBAD-Myc-His A(Invitrogen) digested with NcoI and BglII, enzymes having compatible cohesive ends with BspHI and BamHI.

Plasmid pEAW1033 (Ref ΔN76 in pBAD) was constructed using pEAW685 [25] as a template in a PCR with one primer consisting of a NcoI site followed by bases 229–245 of the *ref* gene. The sequences for aa 77,78,79,and 81 were changed for better codon use. The other primer consisted of a BamHI site, followed by bases 561–544 of the *ref* gene. The PCR product was digested with NcoI and BamHI and ligated into pBAD-Myc-His A digested with NcoI and BglII. Plasmid pEAR12 (Ref ΔC110 in pBAD) was constructed using plasmid pEAW584 as a template for PCR with the same upstream primer used to make pEAW974 and a downstream primer consisting of a BamHI site, a stop codon, and the codons for bases 228–197 of the *ref* gene. The procedure for making pEAW974 was followed. Plasmid pEAR21 (Ref H134/153A in pBAD AKA pRef^nuc-^) was constructed using standard methods to sequentially change the CAC bases at 718–720 (His) at amino acid 134 and the CAT bases at 775–777 (His) both to GCG (Ala).

Plasmid pEAW427 (RbsR in pBAD) was constructed by using E. coli MG1655 DNA as the template in a PCR with one primer consisting of a NcoI site followed by bases 4–23 of the *rbsR* gene, and the other primer consisting of an EcoRI site followed by bases 1243–1226 of the *rbsR* gene. The PCR product was digested with NcoI and EcoRI and was inserted into pBAD-Myc-His A digested with the same enzymes.

### Strain construction

All strains are derivatives of E. coli K-12 and are listed in Table 1. EAR2 was constructed by lysogenizing MG1655 with P1Cm C1.100 produced from strain BR4666. Briefly: 100 μL of P1Cm C1.100 produced from BR4666 was incubated with 200 μL MG1655 overnight culture and 2 μL 1 M CaCl_2_ (5 mM final concentration, necessary for absorption of phage) for 30 minutes at 30°C (to prevent induction of temperature-sensitive phage) without shaking. Then, 1 mL LB and 100 μL 1 M sodium citrate were added to prevent superinfections of the bacteria and the culture was allowed to recover at 30°C for 30 minutes with agitation. Cells were pelleted, resuspended in 100 μL LB, and plated on a LB plate with chloramphenicol for selection of lysogens. Plates were grown at 30°C overnight.

EAW195 is *E*. *coli* MG1655 with P1Cm C1.100 produced from strain BR4666 with *ref* deleted. EAW195 was constructed using a variation of the method in [95]. pEAW507, a plasmid containing a mutant FRT-*KanR*-wt FRT cassette, was used as a template in a PCR. The primers consisted of the 51 bases before the promoter of the *ref* gene +21 bases before one FRT, and the 51 bases after the stop of *ref* +21 bases after the other FRT. The PCR product was electroporated into EAR2/pKD46, and a kanamycin resistant colony was selected. DNA from this colony, designated EAW195, was used as a template in a PCR to confirm the presence of the FRT-*KanR*-FRT replacing the *ref* gene on the lysogen.

EAW20 is MG1655*ΔrecA* and was constructed using a variation of the method in [95]. A plasmid containing the 200 bases upstream of the start of the *recA* gene, followed by a FRT-*KanR*-FRT cassette, and the region starting 6 bases downstream of the stop of the *recA* gene through the *recX* gene, was constructed. This plasmid, designated pEAW413, was used as a template in a PCR with an upstream primer consisting of bases 84–64 before the start of the *recA* gene, and a downstream primer consisting of the 10 bases after the stop of the *recX* gene, and the last 8 bases of the *recX* gene. The PCR product was electroporated into MG1655 containing the plasmid pKD46. A kanamycin resistant colony was used as template in a PCR, and the product was sequenced to confirm the presence of the FRT-*KanR*-FRT cassette replacing the *recA* gene.

EAW28 is EAW13 transduced to *lexA3* (non-cleavable *lexA* variant) with P1 grown on strain SS938 (gift from Steven Sandler, *E*. *coli* JC13509 *del(recA-srl)306*::*Tn10 malE*::*cat lexA3*.

Strains EAR15, 16, and 17 were constructed using standard methods by transforming plasmid pEAW903 into chemically competent MG1655, EAR2, and EAW195, respectively and selecting for ampicillin-resistant colonies.

Strains EAR86, EAR87, EAR88, EAR120, EAR121 and EAR123 were constructed using standard methods by co-transforming chemically competent MG1655 with the plasmids indicated in Table 1 and selecting for colonies both ampicillin and chloramphenicol resistantance.

Strains EAR61, EAR62, EAR73, EAR98, EAR104, and EAR105 were constructed using standard methods by transforming the plasmids indicated in Table 1 into MG1655 and selecting for ampicillin-resistant colonies. Strains EAR64, EAR65, EAR69, EAR70, EAR74, EAR75, EAR77, EAR78, EAR79, were constructed similarly, but using plasmids and background strains indicated in Table 1.

### Survival curves with drug or arabinose added at 5x10^5^ CFU/mL

Drug concentrations used were all at the MIC as determined experimentally using standard methods [97] with *E*. *coli* MG1655: ciprofloxacin used at 8 ng/mL, mitomycin C used at 5 μg/mL, trimethoprim used at 0.5 μg/mL. Independent colonies were grown overnight at 30°C. Cultures were diluted 1:100 in fresh LB and grown at 30°C until OD_600_ was at least 0.35. Cultures were adjusted to OD_600_ = 0.35 with LB (approximately 1x10^8^ CFU/mL), then diluted 1:200 in LB, to give a density of approximately 5x10^5^ CFU/mL. Each culture was split in two, and drug was added to a final concentration as indicated to one of the aliquots. For experiments testing the effect of Ref expression from a plasmid, 1% L-arabinose was also added to all cultures (including empty vector controls). Immediately after addition of arabinose and/or drug, samples were removed and serially diluted 1:10, plated on LB plates supplemented with appropriate selection drugs, and grown overnight at 30°C. The rest of the culture was incubated at 30°C overnight, with samples withdrawn periodically, diluted, and plated as before. Reported data are the average and standard deviation of at least three biological replicates.

### Cell lysis by phage (induced with ciprofloxacin)

Independent colonies of strains EAR2 and EAW195 were picked from freshly streaked plates and grown overnight at 30°C. Cultures were diluted 1:100 in fresh LB and grown at 30°C until OD_600_ was near 0.4. Cultures were adjusted to OD_600_ = 0.3 with LB and 200 μL of each culture was pipetted into two individual wells of a clear 96 well plate, and 10 μL ciprofloxacin was added to a final concentration of 4 ng/mL. A prewarmed plate reader was used to incubate at 30°C and shake while the OD at 595 nm was read every 10 minutes. Reported data is the average and standard deviation of five biological and two technical replicates.

### Cell lysis by phage (induced with a temperature shift)

Strains EAR2 and EAW195 were diluted 1:100 in fresh LB and grown at 37°C until OD_600_ was no more than 0.4. Then, 100 μL of each culture was pipetted into three separate wells on a clear 96 well plate and 100 μL of prewarmed LB (50°C) was added to each well and the plate was immediately placed in a prewarmed (42°C) plate reader. The plate was shaken and incubated at 42°C and the OD at 595 nm was read every two minutes. Reported data is the average and standard deviation of three biological and three technical replicates.

### rt-qPCR

Three independent overnights of EAR2 were grown at 30°C. Overnight cultures were diluted 1:100 in fresh LB plus 5 mM calcium chloride. Cultures were grown on an orbital shaker at 180 rpm at 30°C until the OD at 600 nm was approximately 0.4. An equal volume of fresh LB prewarmed to 50°C was added to each culture and the temperature of the incubator was shifted to 42°C to induce the lytic cycle of the phage. Immediately, a 1 mL sample was taken from each culture and mixed with 2 mL RNA protect Bacteria Reagent (Qiagen) and processed according to manufacturer literature. Additional samples were removed from the main cultures every 10 minutes following the temperature shift until the cultures lysed (became clear), then one additional sample was taken. Samples were treated as above. Cells were lysed and total RNA was isolated using Protocol 7 from the RNeasy Mini Kit (Qiagen). Total RNA was treated with TURBO DNase (Life Technologies) for one hour at 37°C. DNase was removed from the samples using the “RNA Cleanup” protocol from the RNeasy Mini Kit. cDNA was made from 1 μg total RNA using the Superscript III First-Strand Synthesis System (Life Technologies) and 1 μL cDNA was used as a template in qPCR with the 2x SYBR Select Master Mix (Life Technologies), according to manufacturer literature. Primer pairs used are indicated in Table 2. *CysG* was used as a constitutively expressed reference gene. Each cDNA sample was amplified in triplicate, and the resulting C_T_ values for each technical replicate were averaged. Data were analyzed using the ΔΔC_T_ method as described in [56], where ΔC_T_ was determined by normalizing target gene expression to *cysG* expression and ΔΔC_T_ was determined by normalizing the normalized target gene expression at time = t to normalized target gene expression at time = 0. Reported are the average fold-changes from time = 0 of three biological replicates for *ref*. For all other genes, all timepoints with a fold-induction within 20% of the maximum fold-induction for each biological replicate were averaged and the standard deviation is reported.

**Table 2:** Primers used in this study.

Target gene	Upstream primer	Downstream primer	Ref
*ref*	CCGCAAGTGGCAGAAGGC	CTGTTTAGCGATACGGCGGTC	This work
*ban*	GAAAATGCCGCAGTTCGTAAC	TATTCTGCTTGTCCAACAGAATACCC	“
*ssb*	GCGGTGTAAACAAAGTCATCCTG	AACCTTTTCGTAAATACTCACTCGC	“
*dmt*	TCCTTCCTCAAACCGACAGTTATC	GGATTCTCCAGGCCACCTTTC	“
*kilA*	TGCGGATATGAATATATCAAACCTTC	GTCCGCTCGCTCTGTGTAGAG	“
*humD*	TTGAAAGCCGAATTTCTCTTGAT	AACCGGAGTAAGCGAGGAATC	“
*phd*	CGCGGCAACCTTTCTGAA	GGTGTCAAACAGGGATGCAAA	“
*hot*	TATTGCAGCTAAAAGTCAGGAAGAAC	CTTTTGGCAACTGGAGGCTT	“
*parA*	AAAATCACATACGCTTCGCTGTAG	CGAACGAGTTTTACCAGGTCTATG	“
*cysG*	TTGTCGGCGGTGGTGATGTC	ATGCGGTGAACTGTGGAATAAACG	[98]
EMSA			
Ref5’reg	GTTTGGCGAACTCTTGGGTAAG	CGATCGCTCGTTCTCTGGCT	This work
bglB	CGTTAAATCTATCACCGCAAGGG	TTGCTGAAAGCGTTTAATCG	“
GC	ACAGCATCGCCAGTCACTAT	AGCAGCCCAGTAGTAGGTTG	“
ddPCR			
atpH	CCGAGGTAACCAAAAACGAA	TTTCAGCCATAACCCGAATC	This work
bglB	GCTGTCTTTTGCGCCTAATC	GCTGTCTTTTGCGCCTAATC	“
ref	AAGAGCGGGTTTGTATTCCG	TGCGGCTCAATTATAGCAATCA	“

### Lysogenization efficiency

Overnight cultures of *E*. *coli* MG1655 were diluted 1:100 in fresh LB and grown at 37°C until OD_600_ was at least 0.4 (but no more than 0.6). Cultures (500 μL) were washed twice with CM buffer (5 mM CaCl_2_ and 10 mM MgSO_4_) and resuspended in 500 μL CM buffer. Phage prepared above (10 μL) were diluted with 90 μL LB. One tube of each phage was prepared for each biological replicate. MG1655 in CM buffer (200 μL) was added to each phage tube and incubated for 30 minutes at 30°C without agitation. Then, 1 mL LB and 100 μL 1 M sodium citrate were added and cultures were allowed to recover at 30°C for an additional 1.5 hours. Samples were washed with 200 μL M9 buffer twice and resuspended in 200 μL M9 buffer. Cell suspensions were serially diluted 1:10 and spotted on an LB plate to measure viability and an LB plate supplemented with 25 μg/mL chloramphenicol to measure P1 lysogens. Plates were incubated overnight at 30°C and colonies were counted the next morning. Lysogenization efficiency was calculated as CFU/mL chloramphenicol-resistant colonies divided by CFU/mL viable colonies and average and standard deviation of at least three biological replicates is reported.

### Electrophoretic mobility shift assay

The 5' regulatory region of the *ref* gene was PCR amplified from strain EAR2 using primer pair ref5’reg. The high affinity region of bglB was amplified from pMK110 [99] using primer pair bglB. A GC rich region likely to not bind DNA was amplified from pBR322 using primer pair GC. All primer sequences are found in Table 2. DNA was either gel purified or spermidine precipitated using standard methods. 200 pM DNA was 5'-end labeled with [γ-32P]ATP using PNK (New England Biolabs). EMSA was performed as described in [99] and run on a 4% polyacrylamide gel.

### ChIP and digital droplet PCR

Cells for immunoprecipitation were grown to mid-log and lysis was induced as in rt-qPCR experiments, except that the starting culture volume was 200 mL. Immediately after the addition of 200 mL prewarmed media, a 100 mL sample was withdrawn and 10 mM sodium phosphate and 1% formaldehyde were added and the sample was shaken for 5 min at 37°C to crosslink proteins DNA. The reaction was quenched with 100 mM ice cold glycine for 30 minutes on ice. Another 100 mL aliquot of cells was removed and processed 30 minutes after the temperature shift. Cells were harvested, washed with PBS, and frozen at -80°C. H-NS and bound DNA was immunoprecipitated with an H-NS antibody and processed as described in [100]. DNA present at different time points was quantified with the QX100 digital droplet PCR (Bio-Rad). PCR mixes contained 1x EvaGreen supermix, 1:1000 dilution of IP DNA, and 250 nM of each primer to amplify different regions of the *E*. *coli* and P1 phage genomes. Input DNA was run alongside as a control for total amount of DNA present. Three primer sets (Table 2) were used for each IP sample. Signals for *ref* were adjusted for input DNA at the 30 min timepoints to account for increases in the amount of P1 phage DNA compared to *E*. *coli* DNA. IP DNA amounts for *atpH* and *ref* were normalized to IP DNA for *bglB*. Each biological replicate was amplified in technical quadruplicate. Error bars represent the standard deviation of the data.

### SOS response assays

Assays were performed as in [94]. Briefly, overnight cultures were diluted 1:100 in fresh LB and 200 μL was added to the wells of a black-walled, clear-bottom 96 well plate. The plate was incubated at 30°C and shaken orbitally on a plate reader. After 3 hours of growth, lysogens (Fig 4A) had 20 μL ciprofloxacin (diluted in water) added to a final concentration of 8 ng/mL. Protein expression strains (Fig 4B) had 20 μL arabinose added to a final concentration of 1% (instead of ciprofloxacin) to induce protein expression. Wells that did not receive ciprofloxacin or arabinose had 20 μL of water added. The plate was incubated at 30°C shaken orbitally. Every 10 minutes, the plate was briefly shaken linearly and the optical density (OD) at 600 nm and fluorescence at 509 nm (excitation at 474 nm). All values were blank-corrected by subtracting out the average OD or fluorescence value of the blank wells at each timepoint. This resulted in some negative fluorescence values, particularly for cultures that were not undergoing SOS. All fluorescence values were converted to positive numbers by adding a constant to every reading. Fluorescence is reported as the change in fluorescence from time 0. Error bars represent the standard deviation of the data.

### Survival curves with arabinose added at 1x10^8^ CFU/mL

Cultures were grown to OD_600_ = 0.35 as in “arabinose survival curve at 5x10^5^ CFU/mL” experiments. Each culture (approx. 1x10^8^ CFU/mL) was split in two, and L-arabinose was added to a final concentration of 1% to one of the aliquots. Immediately, 100 μL of each culture was removed and serially diluted 1:10, plated on LB plates with appropriate selection antibiotics, and grown overnight at 30°C. The rest of the culture was incubated at 30°C for four hours, with 100 μL samples withdrawn periodically. These samples were diluted and plated as before. Reported data are the average and standard deviation of at least three biological replicates.

### Microscopy of cells expressing Ref variants

Cultures were grown at treated as in “arabinose survival curve at 1x10^8^ CFU/mL” and were allowed to grow at 30°C for four hours (or overnight) after arabinose treatment. A 5 μL aliquot of each culture was removed and incubated with an equal volume of 10 μM DAPI for 5 minutes at room temperature. Then, 1–2 μL of the mixture was placed on a 1.5% agar pad, allowed to soak in, and flipped on to a glass microscope slide. All pictures were taken within a half hour of mounting.

All the images were acquired on a commercial Olympus IX83 inverted microscope equipped with an Olympus UPLSAPO60XS, 1.42 NA silicone oil immersion objective and Hamamatsu C11440 charge-coupled device (CCD) camera operated by a personal computer (PC) running MetaMorph for Olympus software. Brightfield images were obtained using an X-Cite 120 halide arc lamp (Lumen Dynamics Group, Inc.) and Olympus IX3-LWUCDA motorized long working distance (LWD) condenser. The fluorescence excitation for DAPI was performed using the Olympus standard components, and the emission for DAPI was obtain using a 5060C-OFF-Zero (-ZERO pixel set mounted in cube, Semrock) filter set. Contrast of images was adjusted for publication using ImageJ or Adobe Photoshop. Cell length measurements were obtained using the MicrobeTracker plugin for Matlab [66] and were converted to μm using the conversion factor 0.1083 μm/pixel.

### Circular ssDNA nuclease assay

The circular ssDNA from bacteriophage M13mp18 (7249 nucleotides) was prepared essentially as described [101]. The native *E*. *coli* wild type RecA, *Neisseria gonorrhea* RecA, *Staphylococcus aureus*, *Pseudomonas aeruginosa* RecA, and WT Ref proteins were purified as described previously [25, 102–104] (*S*. *aureus* RecA with concentrataion was a generous gift of S. Lusetti), and the concentrations were determined by absorbance at 280 nm using extinction coefficients of 2.23 × 10^4^ M^−1^ cm^−1^ (*E*. *coli* RecA), 2.23x10^4^ M^-1^ cm^-1^ (*Pseudomonas* RecA), 2.49x10^4^ M^-1^ cm^-1^ (*Neisseria* RecA), and 2.85x10^4^ M^-1^ cm^-1^ (P1 Ref). The reactions were carried out as previously described [26], but 100 nM Ref protein was used.

## Supporting Information

S1 Fig SOS induction via Ref expression (1% arabinose) in the presence of ciprofloxacin (8 ng/mL). (TIF)

S1 Table Significant p-values (<0.0001) not depicted in figures/figure legends. (DOCX)
