# Supplementary figures and images for: P1 Ref Endonuclease: A Molecular Mechanism for Phage-Enhanced Antibiotic Lethality
Source: PLoS Genet. 2016 Jan 14;12(1):e1005797. doi: 10.1371/journal.pgen.1005797 (PMC4713147; doi:10.1371/journal.pgen.1005797)

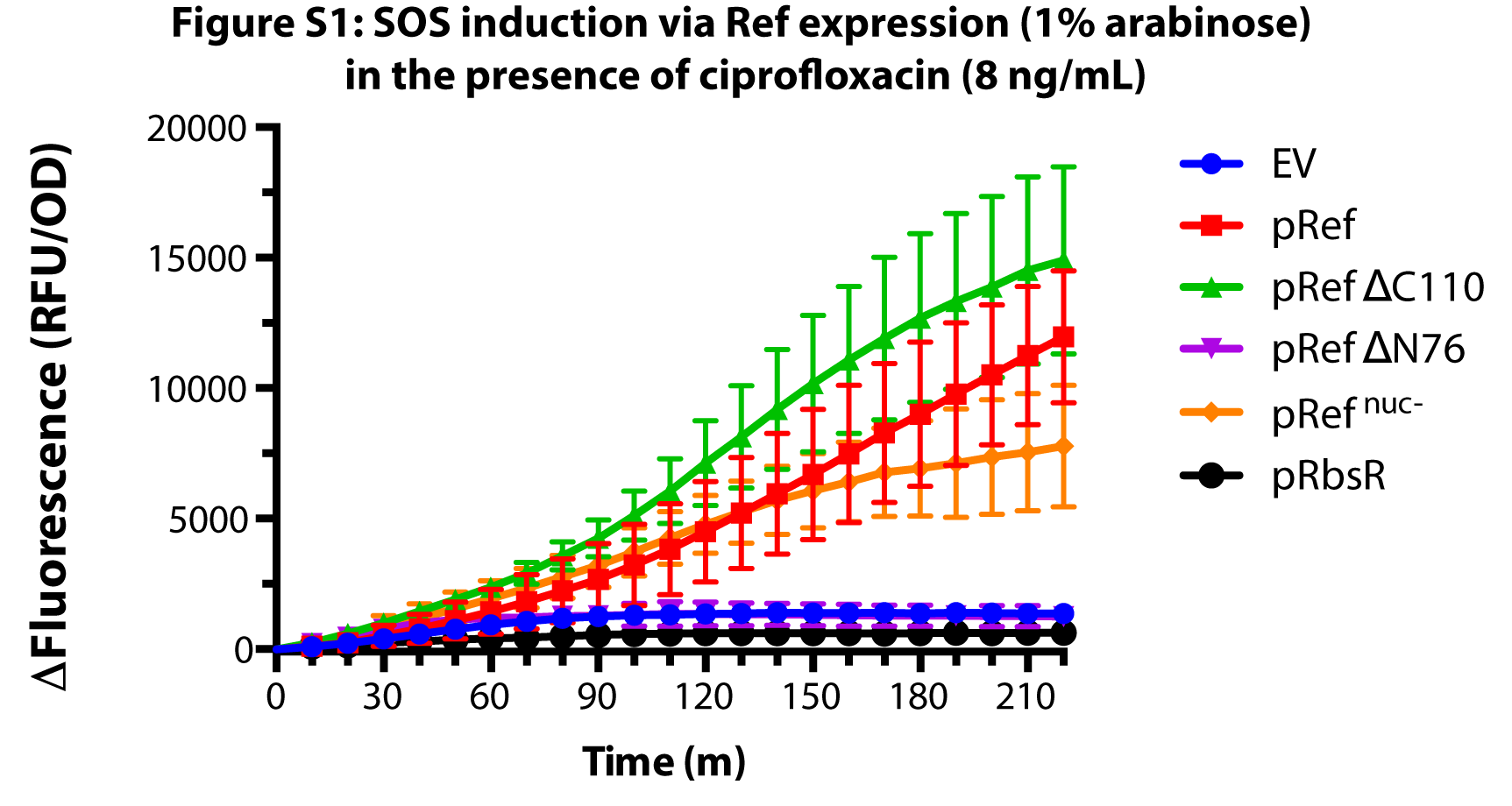

Supplement: S1 Fig — (TIF) [file pgen.1005797.s001.tif]
