# Supplementary material for: P1 Ref Endonuclease: A Molecular Mechanism for Phage-Enhanced Antibiotic Lethality
Source: PLoS Genet. 2016 Jan 14;12(1):e1005797. doi: 10.1371/journal.pgen.1005797 (PMC4713147; doi:10.1371/journal.pgen.1005797)
Supplement: S1 Table — (DOCX) [file pgen.1005797.s002.docx]

S1 Table: Significant p-values (<0.01) not depicted in figures/figure legends

| Figure | Condition | Condition compared | P-value <0.0001 at these timepoints |
| --- | --- | --- | --- |
| 5C | WT pRef | WT EV | 2h, 4h |
|  | WT ΔC110 | WT EV | n.s. |
|  | *ΔrecA* pRef | *ΔrecA* EV | 2h, 4h |
|  | *ΔrecA* ΔC110 | *ΔrecA* EV | 2h, 4h |
| 6A | EV | pRef | 6h (0.0007) 8h, 20h |
|  | EV | ΔN76 | n.s. |
|  | EV | RbsR | n.s. |
|  | EV | ΔC110 | 2h, 4h, 6h, 8h |
|  | EV | Refnuc- | 8h, 20h |
|  | *ΔrecA* EV | *ΔrecA* pRef | 8h, 20h |
